# A flexible automated magnetic microrobot assembly system

**DOI:** 10.1007/s12213-026-00204-y

**Published:** 2026-03-19

**Authors:** Oliver J. Shindell, Aaron C. Davis, David J. Cappelleri

**Affiliations:** 1 School of Mechanical Engineering, Purdue University, West Lafayette, IN USA; 2 Weldon School of Biomedical Engineering (By Courtesy), Purdue University, West Lafayette, IN USA

**Keywords:** Microassembly, Microrobotics, Micromanipulation

## Abstract

This paper presents a novel system for flexible automated fabrication of microrobots with embedded permanent magnets, and for the loading of liquid therapeutic drugs and sealing with thermally sensitive wax. Microrobots featuring embedded magnets are more controllable and observable, and are capable of tasks requiring higher forces. In this system, a micromanipulator controls tweezers, and stepper motors actuate a four-stage system that executes different assembly steps. A syringe pump is used to fill drug delivery microrobots, and a wax seal is applied with a brush made from heated copper wires. This brush is capable of efficiently applying an even wax coating to drug delivery robots, sealing the contained therapeutics inside. Vision-based feedback from an overhead microscope camera ensures precise embedded magnet assembly through a combination of image processing algorithms. A single drug delivery robot can be assembled in 192.77±48.28 seconds (mean±standard deviation, n=13). Drug loading and sealing takes 159.38±3.67 seconds (n=16). 100% of seals were found to be hermetic, with an average thickness of 302±25 $\mu$m (n=16). This work advances micro-assembly toward practical medical use by establishing a practical basis for mass production of drug delivery robots.

**Supplementary Information:**

The online version contains supplementary material available at 10.1007/s12213-026-00204-y.

## Introduction

Progress in the field of microrobotics indicates high potential for minimally invasive medicine and tissue engineering [1, 2]. Magnetic microrobots are favored for untethered, precise, and safe *in vivo* control [3, 4]. Additionally, they offer versatility in design and can perform a wide range of tasks [5]. Increasing the amount of magnetic material in a microrobot, and the strength of magnetic material, yields more precise control and a wider range of functionality [6]. An effective way to do this is to embed a strong permanent magnet. An additional benefit offered by this is ease of detection. The size of microrobots prohibits the use of embedded electronics that broadcast signals, so they must be observed by an external source [7]. An embedded permanent magnet can be distinctly observed by an ultrasound system, making detection more accurate [8].

A popular use of magnetic microrobots is targeted drug delivery [9–13]. Phase-change materials such as paraffin and biocompatible waxes are a promising mechanism for controlled drug release, due to their well-defined melting points, chemical stability, and hermetic sealing [14]. Several methods exist to effectively control the melting of wax *in vivo*, such as magnetic fields and focused ultrasound [15, 16]. Using wax-sealed microcapsules has been demonstrated to provide reliable containment and temperature-triggered release in biocompatible environments [17, 18]. The use of a wax seal can also be used to create vacuum chambers that open when certain biological materials are present [19]. 3D-printed magnetic microrobots have incorporated temperature-sensitive wax caps to confine liquid payloads within internal cavities, enabling wireless control of both locomotion and drug release [20, 21].

Drug delivery is an area that benefits from the use of microrobot swarms [22, 23]. Magnetic microrobots are also effective when performing other tasks in large swarms, such as moving larger objects or guiding biological samples [24–27]. Swarms also offer a platform for increased functionality through collective control [28, 29]. For the fabrication of large quantities of microrobots, an automated systems is highly desirable.

Embedding a magnet into a microrobot requires a delicate and precise pick-and-place operation. This sort of contact-based manipulation is a prominent field of research, with work in areas such as assembly [30, 31], grasping delicate objects [32], and manipulating magnetic particles [33]. There is also specific research on how to assemble magnetic robots [34]. To assemble large numbers of microrobots, it is desirable to have a system that works autonomously, with consistency and no human oversight. Presented in this paper is an automated system for permanent magnet embedding, liquid drug loading, and phase-change material-based sealing for microrobots. It employs electro-mechanical actuators and computer vision to autonomously assemble microrobots using contact-based manipulation, removing the necessity for human operation and serving as a basis for scaling production.

**Fig. 1:**
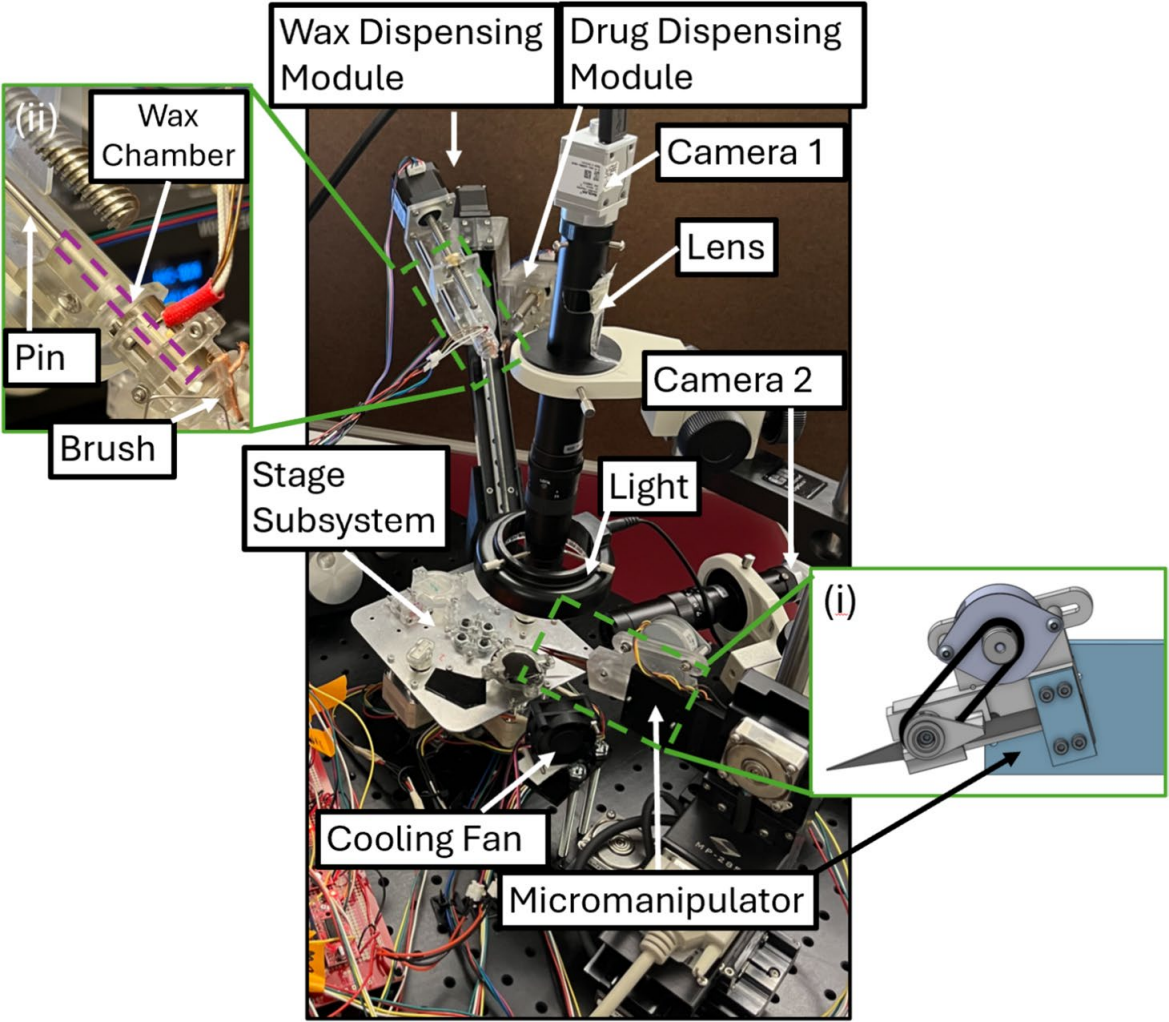
Full labeled view of system. (**i**). Close-up of tweezer actuation system. (**ii**). Close-up of wax dispenser

**Fig. 2:**
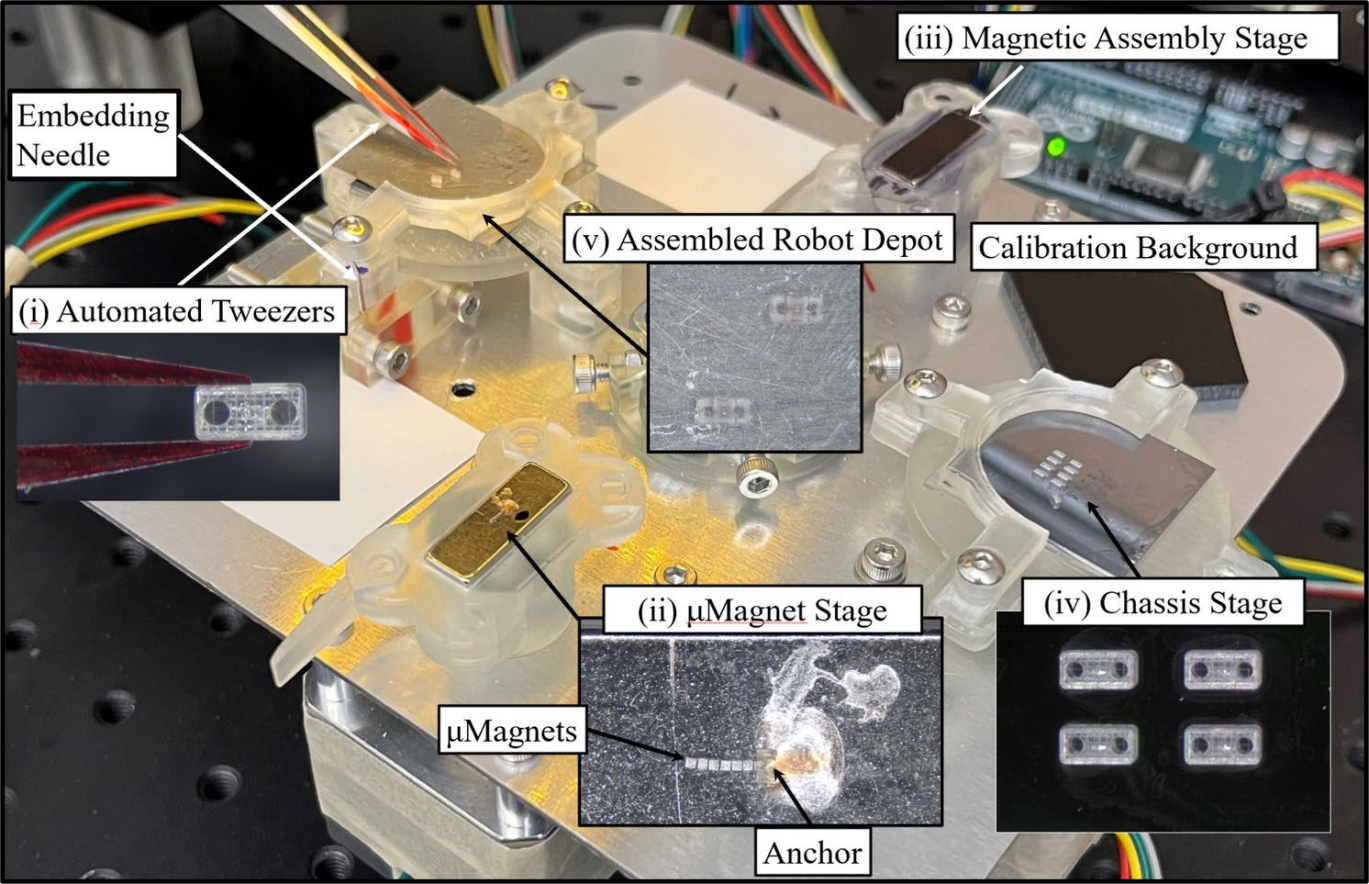
Stage subsystem view. (**i**). Close-up view of tweezers. (**ii**). Close-up of $\mu$-magnet stage. (**iii**). Assembly stage. (**iv**). Chassis stage. (**v**). Depot stage

## System overview

The developed assembly system is shown in Fig. 1. A detailed view of the stage subsystem is shown in Fig. 2, with a close-up of the tweezer tips seen in Fig. 2(i). The stepper motor in Fig. 1(i) opens and closes tweezers (Dumont) using a lead-screw system. This motor, along with the others in the system, is driven by a stepper motor driver. These drivers are controlled by a microcontroller (Arduino). The host computer communicates by serial with the microcontroller, the micromanipulator controller (Sutter MP-285), and the camera (Basler). The micromanipulator (Sutter) tracks its own state as steps in XYZ coordinates. The camera is fitted with a 0.75X magnification lens (Edmund Optics), which is fixed at a height such that all $\mu$-magnets and chassis are in the focus plane. The host computer runs a Python script that interacts with all three peripherals to perform assemblies.

### Stage subsystem

The stage subsystem has eight areas on it, and the entire system is mounted on a stepper motor, which can position each area under the camera. The black and white calibration backgrounds are used to obtain clear images of the tweezers, which are used to relate the state of the micromanipulator to the video feed from the camera. Placing them below the focal length of the lens yields a sharp image of the tweezers. The robot depot is used to store assembled robots. It typically holds a nickel substrate covered with a small amount of resin, which ensures that robots adhere to the depot rather than the tweezers upon release. For drug loading and wax sealing, this is exchanged for a substrate with larger vertical features that allow the microrobots to be forced off the tweezers. Also on the robot depot is an embedding needle, which is used to push magnets deeper inside robots when necessary. The chassis stage holds empty chassis on a substrate. Both the chassis stage and robot depot have a semi-circular fixture that secures the substrate placed on them. Placing different substrates on these stages can change the height of the surface, so set screws are used to keep all the stages at the same height.

Both the $\mu$-magnet stage and assembly stage are N52 magnets, poled through their length, that are pressed into SLA printed pieces set-screwed to stepper motors. The $\mu$-magnet stage holds the supply of $\mu$-magnets to be embedded into chassis. Figure 2(ii) shows the features that enable magnets to be isolated from each other; this process is explained in Section 3.2. The anchor is made with two-photon polymerization [35], with an embedded $\mu$-magnet the same size as those for assembly. It is secured in direct contact with the stage by an adhesive.

**Fig. 3:**
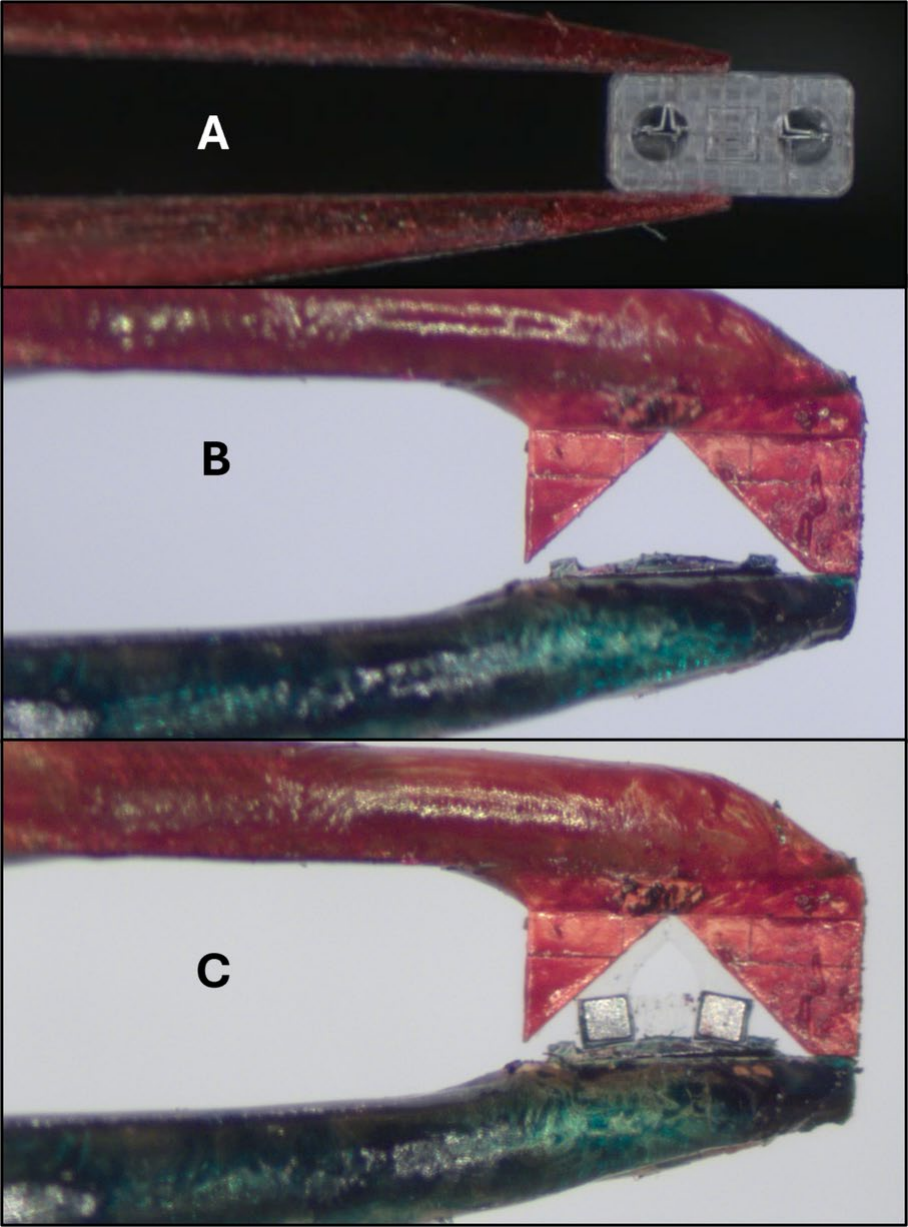
(**A**) Tweezers holding a tumbling robot chassis. (**B**) Tweezers with modified tips. (**C**) Tweezers with modified tips holding an assembled gripper robot

The tweezers are fixed at a constant $15^{\circ }$ angle with the XY plane. Different tweezers can be removed and replaced. Shown in Fig. 3 are modifications made to tweezers. These modifications are made to improve visibility, or to tweeze chassis that are too fragile, have excessively complex geometry, or are too flexible to be grasped by the tweezer tips. Figure 3(A) shows a rectangular chassis grasped by tweezer tips. The tips are colored red for high contrast. In Fig. 3(B), pieces made using two photon polymerization are affixed to the tweezer tips. They are tailored to accommodate the chassis to be grasped. Figure 3(C) shows the grasping of a microrobot that consists of two sides joined by a flexible spring-like structure. The smooth distribution of pressure mitigates mechanical destruction or deformation of the chassis and ensures that flexible parts are sufficiently constrained. Such a method of grasping has also proved effective in maniuplating soft PDMS-based chassis with this system. Further information on the microrobots shown in this figure can be found in [21, 36].

**Fig. 4:**
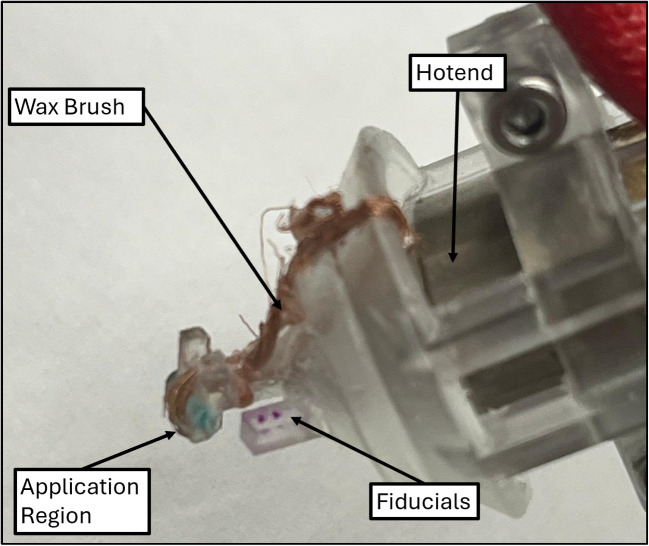
Close-up of wax applicator. The dispensing needle has been removed to provide a better view of the wax applicator, but would normally be in a set position relative to the fiducials and be visible from this angle

### Drug loading and wax sealing

The systems for wax and drug dispensing can be seen in Figs. 1 and 4. Both of these systems operate using linear stepper motors, and are affixed to another linear stepper motor that moves them in and out of the field of view of the overhead camera. The wax dispenser works by pushing wax through a hotend (Creality), which then collects on copper wires. Figure 1(ii) shows a pin pushing into the hotend using a lead screw mechanism. The wax chamber is loaded with powdered wax (n-Heneicosane 98%, ThermoFisher), and when the pin advances, liquid wax is expelled through the hotend. This wax has been shown to have a stable melting point and to be biocompatible [21]. Figure 4 shows the wax brush which is used to apply wax to the tops of microrobots. It is made of bare copper wires that are held in tension over the applicator region. When the wires are coated with liquid wax, a microrobot can be touched to the underside of the applicator region to apply a wax coating to the top surface. The region of the brush that is used for seal application is about 1.5mm by 1 mm. This method of waxing allows a high degree of control over the coating process. A fan is set up to expedite the cooling and solidification of the wax seals, as seen in Fig. 1. For the drug loading, a simple lead-screw based syringe pump is used to control the dispensing. The needle is a 32G dispensing tip. It is loaded with a solution of Brilliant Green dye, which is used used here as a stand-in for actual therapeutic drugs. Simple fiducials allow for precise location of the needle and brush.

## System operation

The system requires that materials for assembly be loaded before operation begins. The necessary materials are $\mu$-magnets, chassis, a substrate for the robot depot, liquid therapeutic drugs, and powdered wax. $\mu$-magnets are loaded onto the $\mu$-magnet stage as needed. This stage begins positioned under the camera, oriented as seen in Fig. 2(A). The assembly stage is oriented such that the poling direction of the embedded magnet is opposite that of the $\mu$-magnet stage – in this way it has the same poling direction as the $\mu$-magnet stage once it is under the camera, which makes the release of $\mu$-magnets from the tweezers much more stable. To maximize assembly capacity, chassis are all be printed in the same orientation and centered on the stage. Wax is loaded into wax chamber by pushing the powder into the chamber while the hotend is off. The brush is dipped in liquid wax, once, before being assembled permanently with the hotend. It remains saturated with wax, using the ejected wax from the hotend to remain so.

### Calibration

Calibration is necessary prior to operation. The micromanipulator operates on an XYZ coordinate system of steps, each step being about one micron. Using the profile camera, the Z axis calibration is done by finding the micromanipulator Z state at which the tweezers touch the stages, injection needle, brush, and focal plane. These calibrations are stable until the system is reconfigured, and have a wide margin of error.

The micromanipulator must be able to precisely move the tweezers to any location in view of the camera (only the top view camera is used online). Thus, a relationship must be established between the micromanipulator’s XYZ state and XY coordinates of pixels seen by the camera. This is done in a similar manner to the linear interpolation found in Eqs. 1-2 of [37]. In the system presented in the current work, the calibration is done in two parts—a motion calibration first, followed by a position calibration. This allows the tweezers to be moved to any desired point within the camera’s coordinate system by knowing the micromanipulator state that brings the tweezers to the center of the image and the amount that they will move within the image when the micromanipulator state changes by a certain amount.

For the motion calibration, representing the micromanipulator state as $(X_M, Y_M, Z_M)$and the position of the tweezers in the image as $(X_{twz}, Y_{twz})$ in pixels, we introduce the constants $X_X, Y_X, X_Y,$ and $Y_Y$, which relate change in $(X_M, Y_M)$ to change in $(X_{twz}, Y_{twz})$ with the following equations:

1 $$\begin{aligned} \Delta X_M * X_X + \Delta Y_M * X_Y&= \Delta X_{\text {twz}}, \end{aligned}$$

2 $$\begin{aligned} \Delta X_M * Y_X + \Delta Y_M * Y_Y&= \Delta Y_{\text {twz}}. \end{aligned}$$

**Fig. 5:**
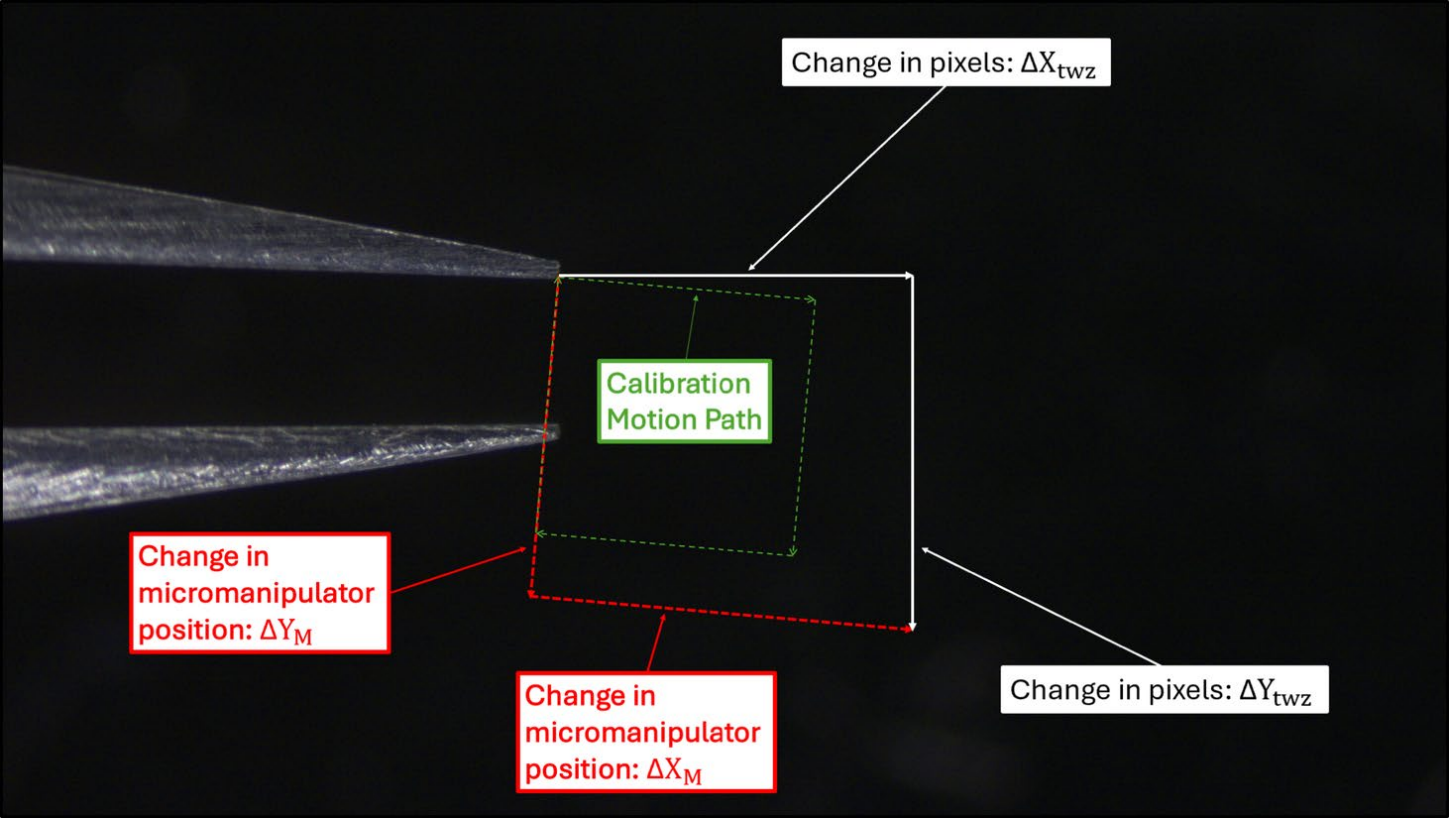
Calibration of tweezer motion to camera coordinates. The green arrows show the path traveled by the tweezers during calibration, a 1mm-side square. The red arrows show orthogonal motions $\Delta X_M$ and $\Delta Y_M$ along the X and Y axes of the micromanipulator. The white arrows show $\Delta X_{\text {twz}}$ and $\Delta Y_{\text {twz}}$, the result of the micromanipulator motion as seen by the camera

The following Fig. 5 shows the coordinate systems of the micromanipulator and the camera, and the calibration done to allow the micromanipulator to move by a given amount within the camera’s coordinate system. By recording the coordinates of the tweezers at several points in both the micromanipulator coordinate system and the camera coordinate system, the values of the constants $X_X, Y_X, X_Y,$ and $Y_Y$ can be calculated by solving for them in Eqs. 1 and 2. Since the micromanipulator records its position in micrometers, this calibration data can also be used to solve to a directionless value of pixels per micron, $P_M$, using Eq. 3.

3 $$\begin{aligned} P_M = \sqrt{X_X^2 + Y_X^2} = \sqrt{X_Y^2 + Y_Y^2} \end{aligned}$$

For the position calibration, the $X_M, Y_M$ that hold the tweezers at some location $X_{twz}, Y_{twz}$ must be found. Let ($X_{cal}$, $Y_{cal}$) be the values of $(X_M, Y_M)$ that position the tweezers in the center of the image, this center being $(X_{cnt}, Y_{cnt})$ in pixels. Applying algebra yields the following equations:

4 $$\begin{aligned} U_X&= X_{\text {des}} - X_{\text {cnt}} + X_Y * Y_{\text {cal}} + X_X * X_{\text {cal}}, \end{aligned}$$

5 $$\begin{aligned} U_Y&= Y_{\text {des}} - Y_{\text {cnt}} + Y_Y * Y_{\text {cal}} + Y_X * X_{\text {cal}}. \end{aligned}$$

6 $$\begin{aligned} X_M&= \frac{X_Y * U_Y - Y_Y * U_X}{X_Y * Y_X - X_X * Y_Y}, \end{aligned}$$

7 $$\begin{aligned} Y_M&= \frac{X_X * U_Y - Y_X * U_X}{X_X * Y_Y - Y_X * X_Y}. \end{aligned}$$

$X_{des}$ and $Y_{des}$ are desired coordinates in the image, and $U_X$ and $U_Y$ are auxiliary variables used to simplify the calculation; they represent no physical quantity. Using these equations, the micromanipulator state that positions the tweezers at a desired location in the image can be found. As with the Z calibrations, this is stable until the system is reconfigured.

**Fig. 6:**
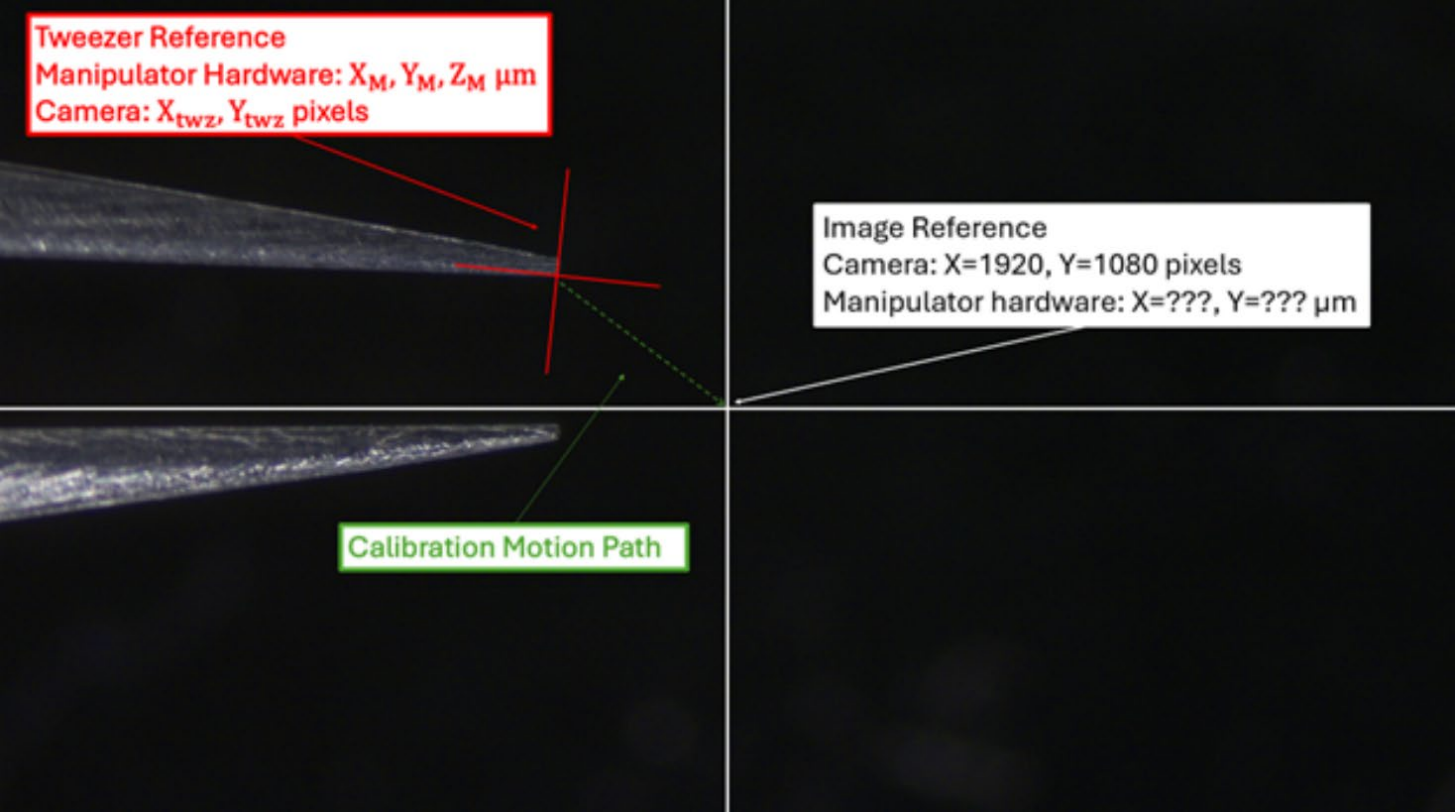
Calibration of tweezers to image center. The current position of the tweezers in pixels is checked with a CV algorithm. Using the motion calibration, the tweezers are moved to the center of the image to determine the appropriate manipulator hardware coordinates at this position in $\mu$m. This is repeated recursively to reduce noise and improve calibration accuracy

Figure 6 shows the position calibration process. The XY position of the tweezer tip relative to the micromanipulator state changes whenever the jaw opening changes or an object is grasped. This necessitates re-evaluating $X_{cal}$ and $Y_{cal}$ online. This is done by detecting the tweezer tip, a process detailed in Section 4C, and using Eqs. 6 and 7 to attempt to move it to the center of the image. Then the values of $X_{cal}$ and $Y_{cal}$ are reset to the XY state of the micromanipulator. This process is repeated until the camera sees less than 2 pixels of motion in one iteration (this number can be modified to make calibration faster but less precise). If the captured images are noisier, it can be difficult for the system to converge to a calibration in this manner, so the movements can be forced to converge to zero if needed by scaling them down over iterations.

**Fig. 7:**
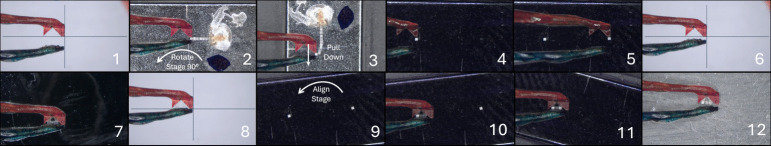
Assembly steps: (**1**) calibration, (**2**) magnet grasping, (**3**) magnet isolation, (**4**) magnet placement, (**5**) second magnet placement, (**6**) calibration, (**7**) chassis grasping, (**8**) calibration, (**9**) magnet alignment, (**10**) magnet embedding, (**11**) realignment and second magnet embedding, (**12**) finished robot release

### Magnet embedding

Loaded with the necessary materials and calibrated, the system can begin operation. A series of images showing the operation steps is seen in Fig. 7. (1) The tweezers are first calibrated over a calibration background. Different background can be used to better contrast with different objects. The jaw distance is also adjusted to a desired value. (2-3) Next, a $\mu$-magnet on the $\mu$-magnet stage is grasped and removed from the group. This is done by rotating the stage while moving the tweezers such that the $\mu$-magnet group remains in line with and attached to the anchor. Rotating 90 degrees orients the tweezed $\mu$-magnet perpendicular to the others, which allows it to be removed easily. (4) The isolated magnet is then placed on the assembly stage. (5) Steps 2-4 are repeated, placing another $\mu$-magnet on the assembly stage. (6) Once all $\mu$-magnets are placed, the tweezers are recalibrated and the jaw opening adjusted. (7) A chassis is taken from the chassis stage, (8) after which another tweezer calibration is performed. If the tweezer tips are unmodified, it is desirable to calibrate based on the grasped chassis rather than the tweezers themselves. (9) After calibration, the assembly stage rotates to orient a $\mu$-magnet for embedding with correct poling. (10) The chassis is then placed onto the $\mu$-magnet, securing it inside. During this placement, the tweezers descend in a helical pattern, correcting for any small alignment errors. (11) This is repeated for the second hole, again re-orienting the $\mu$-magnet to ensure it is poled in the proper direction ($\mu$-magnets are poled in opposite directions for the robot shown in the figure). (12) Finally, the completed robot is released. If there are more robots to be assembled, the system continues.

### Preventing undesirable magnetic interactions

During this process, it is vital that the undesired coupling of micromagnets and the adhesion of micromagnets to the tweezers be prevented. The operation and design of the system incorporates several features to deal with the interaction forces between micromagnets and with the presence of static forces. These occur differently and present unique problems at various stages of the assembly process.

**Fig. 8:**
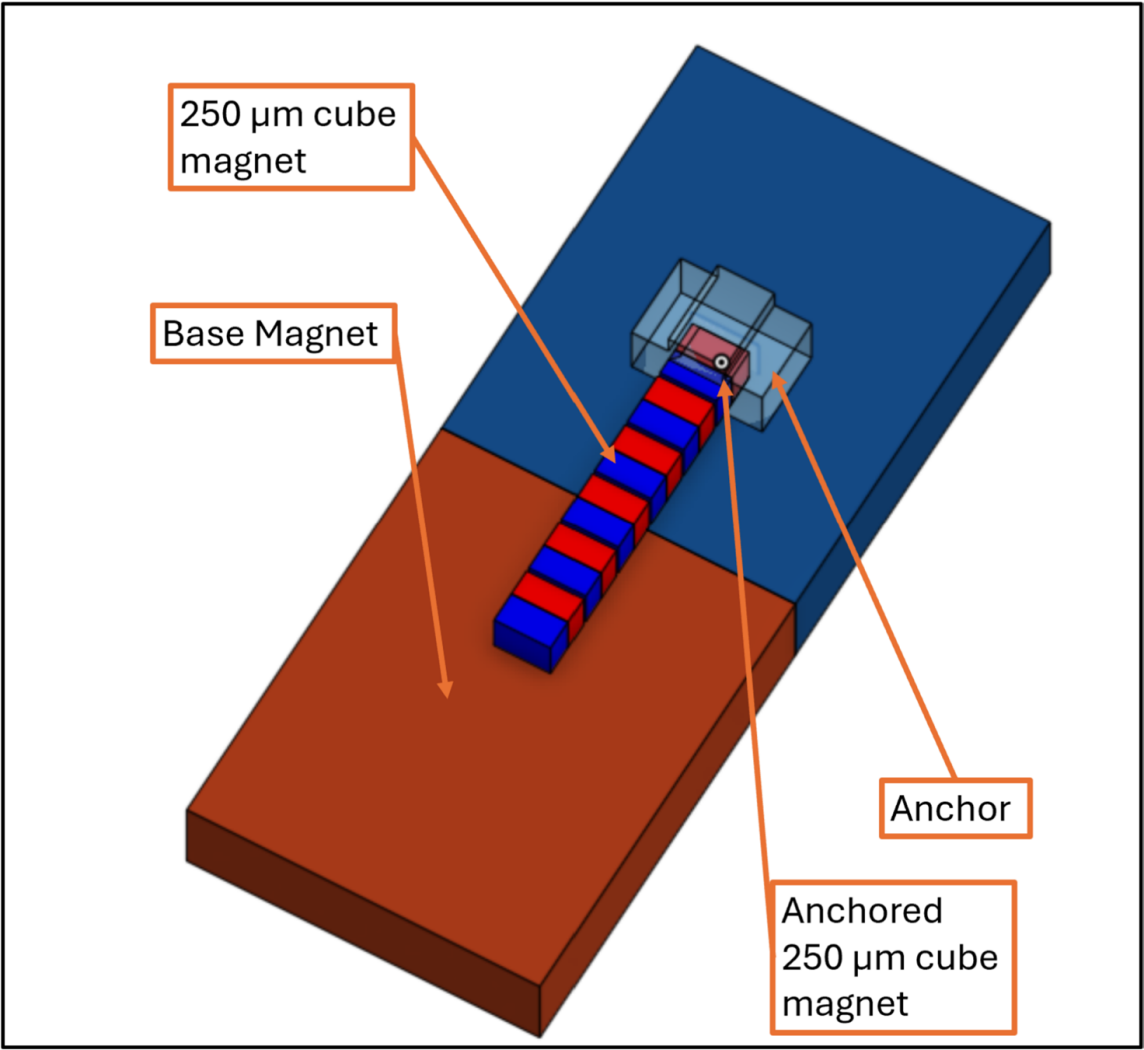
Schematic of micromagnets held in storage with an anchor, on the surface of a larger base magnet

The first step in which micromagnet-micromagnet interactions present a problem is in the separation of a single magnet from a larger group. Figure 8 shows how the micromagnets used for assembly are stored in the system. The line, or chain, of micromagnets allows the system to hold large quantities of magnets in a stable configuration. This works by holding the chain of magnets on top of a large base magnet of equal flux density, as shown in Fig. 8. A single magnet on one end of the chain is permanently fixed in place inside an anchoring fixture and is not used as a component for microrobots.

Storing the micromagnets in this manner is efficient and stable, but removing an individual magnet is difficult. If the un-anchored end magnet is simply pulled off, it may take the rest of the chain with it. Since magnets must be isolated for assembly, it must be ensured that only one magnet is removed from the chain at a time, while the rest remain attached to the anchor. This is done by grasping the end magnet with the tweezers, and then rotating the base magnet, along with the anchor and the remaining magnet chain, 90$^{\circ }$ relative to the tweezers. Doing this breaks the alignment between the grasped magnet and the rest of the chain, allowing it to be separated and isolated. This technique is what is shown in Fig. 7, steps 2 and 3.

**Fig. 9:**
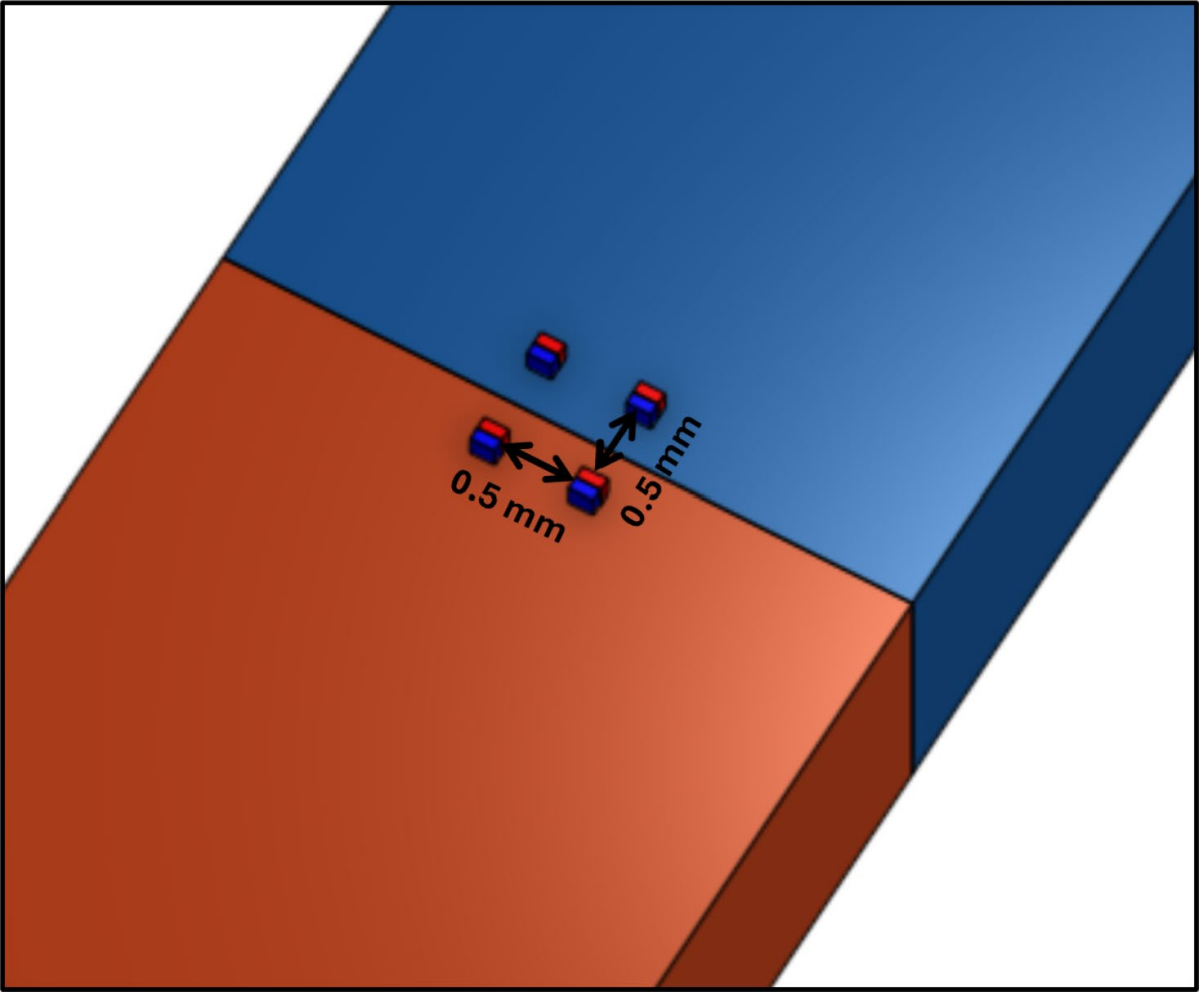
Example layout of multiple micromagnets on the assembly stage. Shown here are 250 $\mu$m-side cube micromagnets, spaced 0.5 mm apart in a square pattern. In this configuration, all four magnets are in field of view but will not snap together. Greater spacing can also be used

Magnetic forces can also have undesirable effects between micromagnets on the assembly stage or during the magnet embedding process, particularly for microrobots with multiple embedded micromagnets. Figure 9 shows how multiple micromagnets can be placed on the assembly stage at the same time. Micromagnets in this configuration are sufficiently far from each other so that they will not snap or stick together. In this configuration, the magnetic fields generated by the micromagnets are small compared to the field generated by the larger base magnet below it. This spacing has been empirically determined.

Two other undesirable behaviors can also occur that must be accounted for. The first is that when the micromagnets are released from the tweezers, they can snap to one of the ends of the base magnet rather than staying relatively in place. This is effectively prevented by ensuring that the micromagnet is properly aligned with the base magnet when it is released. The system is also programmed to check if this occurred and will retrieve another magnet if a failure is detected. Static forces can also occasionally cause the micromagnet to stick to the tweezers. To prevent this, the assembly stage can be rapidly rotated back and forth through a ±10$^{\circ }$ angle, and the resulting changing magnetic field will perturb the micromagnet and cause it to fall from the tweezers onto the assembly stage.

A second issue can occur in instances after one micromagnet has been embedded in assemblies that contain multiple micromagnets. The second micromagnet can sometimes snap to the first one that has already been embedded. Note: the embedded magnets have an interference fit to prevent them from coming back out, so the embedded magnet is never pulled to the un-embedded one. The snapping of the second magnet to the embedded magnet is prevented by using a base magnet of flux density greater than or equal to that of micromagnets and doing the micromagnet embedding directly on the surface of this base magnet. Figure 7 steps 10 and 11 show the process of embedding directly on the surface of the base magnet. The base magnet for the assembly stage is black for greater image contrast, but it is otherwise an identical magnet to the one used in the micromagnet storage stage.

**Fig. 10:**
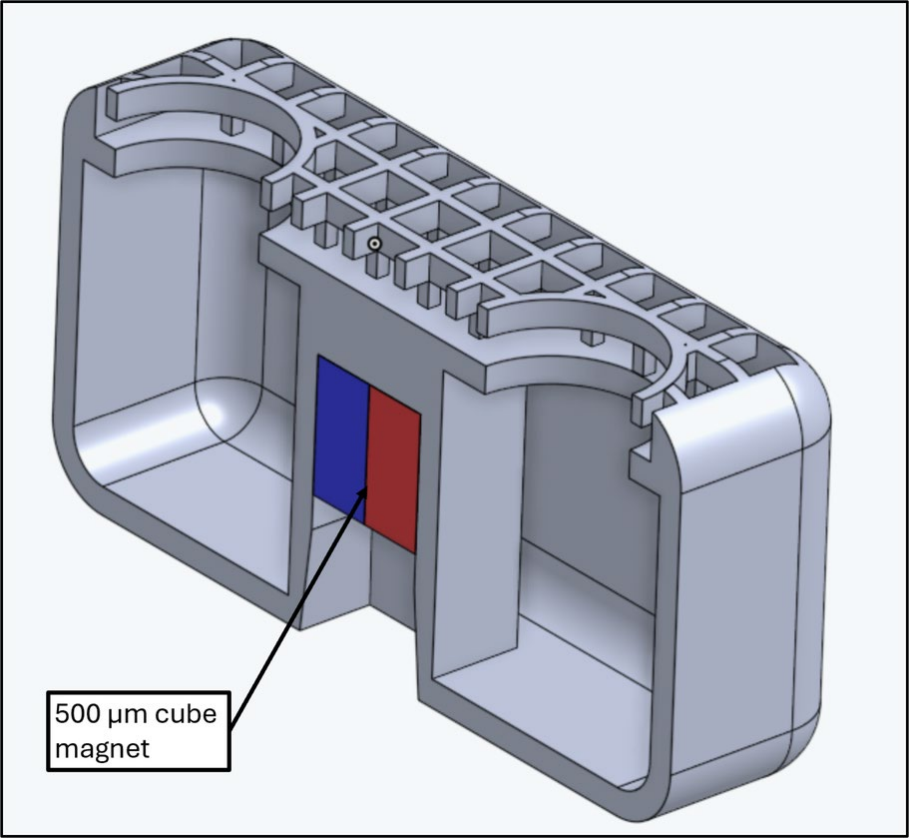
Half section of drug delivery tumblers. Inner cavity holds liquid payloads, top mesh helps secure wax seal, and permanent magnet allows for tumbling locomotion. Circular top ports are used for drug loading and release

### Deep embedding

**Fig. 11:**
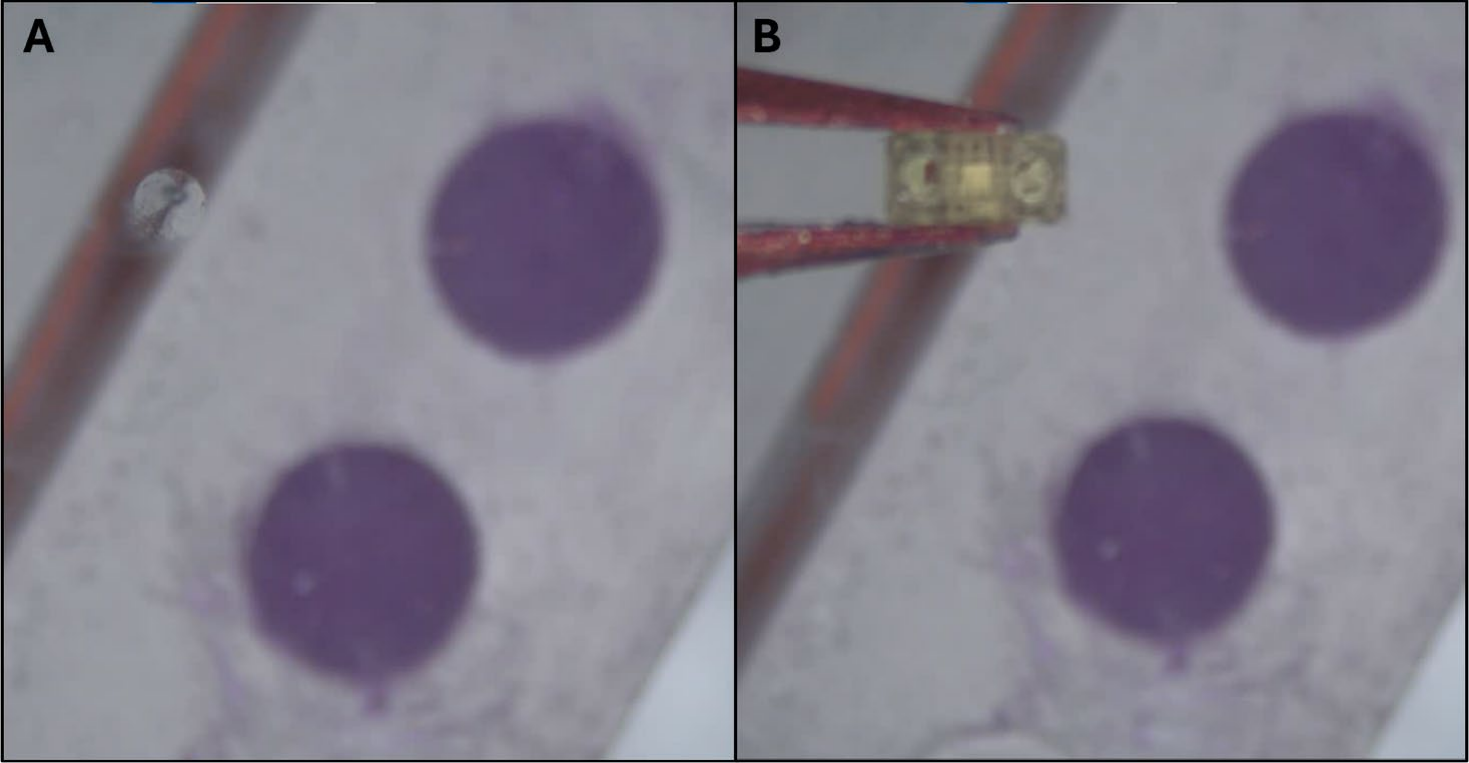
Deep Embedding process. (**A**) top view of the embedding needle. (**B**) tumbler being lowered onto the embedding needle

The tumbling robots used in this study are shown in Fig. 10, and are the same as those found in [20]. This figure shows the half section of a tumbler. The mesh on the top improves the wax coat adhesion. The magnet inside, a 500 or 250 $\mu$m cube, is pushed deeper than the bottom surface of the robot; to achieve this in assembly, the embedding needle is used. After the initial magnet insertion shown in Fig. 7, the partially-embedded magnet is pushed all the way inside by lowering the tumbler onto the embedding needle with the micromanipulator, as shown in Fig. 11. This deep embedding allows the robot to be taller while keeping the center of mass and magnetic moment in the center, which increases the carrying capacity without harming control capability.

**Fig. 12:**
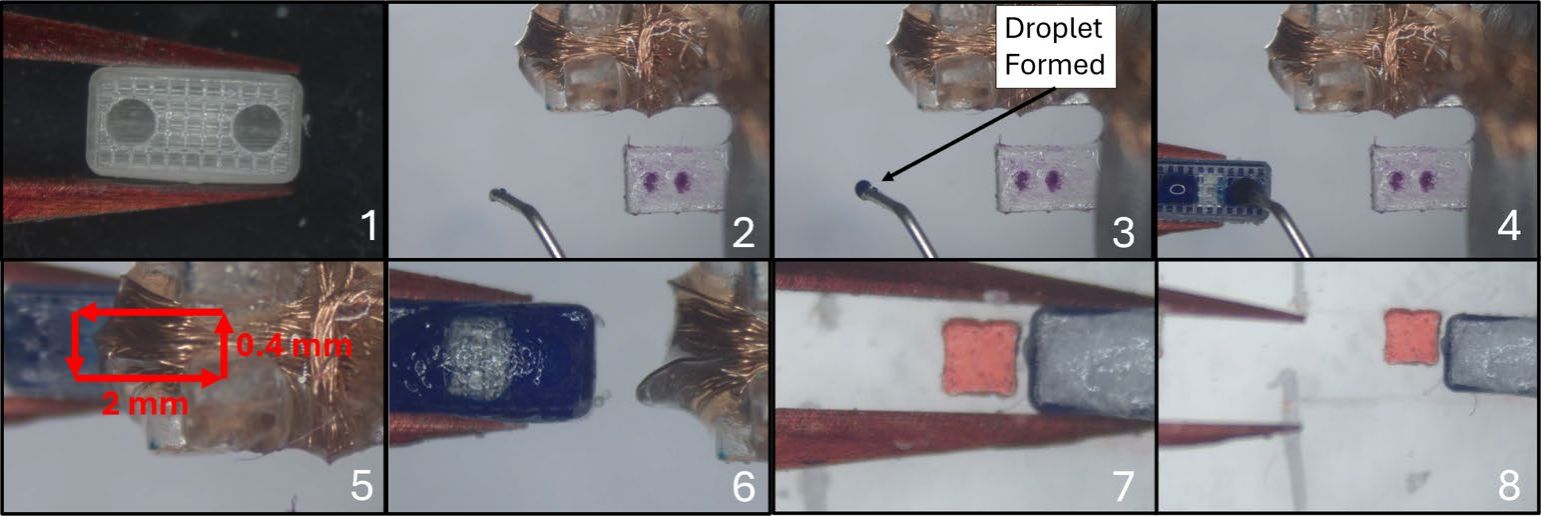
Wax application steps: (**1**) pick up tumbler, (**2**) lower slide stepper, (**3**) prime needle with droplet, (**4**) fill tumbler, (**5**) brush wax onto tumbler in pattern shown for 10 passes, (**6**) remove tumbler from brush, (**7**) move tumbler to depot stage, (**8**) release tumbler using the hardstop

### Drug loading and waxing

The system follows additional steps for the filling and waxing of tumblers after the magnet has been embedded. An outline of the steps followed can be seen in Fig. 12. (1) Once the tumbler is picked up, the loading systems are lowered into the field of view of the overhead camera. (2-3) Before injection occurs, the needle must be primed. This priming is done by advancing the syringe pump until a droplet begins to form on the end (camera feedback ensures a consistent droplet size). This is necessary to expel any air that may collect in the needle, ensuring that a consistent amount of liquid is dispensed into the microrobot. (4) After the tumbler is filled (slightly overfilling the cavity ensures the tumbler will be consistently fully loaded), (5) wax is applied to the top of the tumbler by rubbing it against the brush for 10 passes in the pattern shown. (6-7) The tumbler is removed from the brush and brought to the depot stage. (8) Finally, the tumbler is released by relaxing the tweezer grip, and a hardstop (shown in red) ensures full release.

The main factor that affects the seal properties is the temperature of the wax brush, and to a lesser degree the number of passes of the brush. See Section 5.2 for a full description of the data collected on these effects. Based on this data, running the brush at 85$^\circ$C for 10 passes was chosen as the operating point of the system. The pin and chamber system must be used to replace the wax that is consumed in the coating process.

**Fig. 13:**
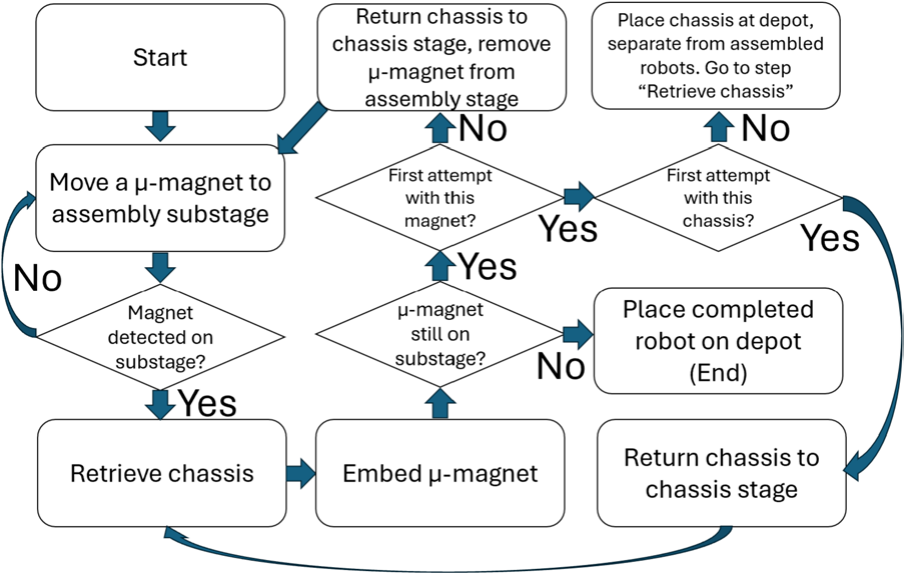
Safety logic flowchart

### Assembly completion and fail-safes

Once the system finishes, a set of assembled robots is left on the robot depot. Completed robots can be washed with iso-propyl alcohol, to remove excess resin from inside the chassis or the substrate and any coloring that may have stuck on the robots during the assembly. Curing with UV light secures the $\mu$-magnets inside.

Manufacturing defects in the $\mu$-magnets or chassis can cause assembly issues. As such, logic is employed to detect and deal with assembly failures when they occur, as shown in Fig. 13. $\mu$-magnet embedding may fail if a chassis hole is too small or a $\mu$-magnet is too large. When this happens, the chassis rotates in the tweezers and needs to be released and regrasped to fix the orientation. If either a $\mu$-magnet or a chassis fails twice, the system deems it as defective and discards it. This ensures that the system works even if a step fails or a part is defective. Some metrics on the frequency of these failures are found in Section 5.3.

## Computer vision and image processing

In order to execute the processes described in Section 3, the system employs a variety of vision algorithms to obtain the positions of magnets, tweezers, and chassis. These algorithms primarily use the OpenCV library available in Python. Because the system is highly stable, it is unnecessary to use continuous image feedback. Rather, the host computer will process a single image, and based on that will send instructions to the microcontroller and micromanipulator to complete assembly steps, the only exception being the iterative calibration of the tweezers. Processing a small number of images allows algorithms to run slowly without major drawbacks.

**Fig. 14:**
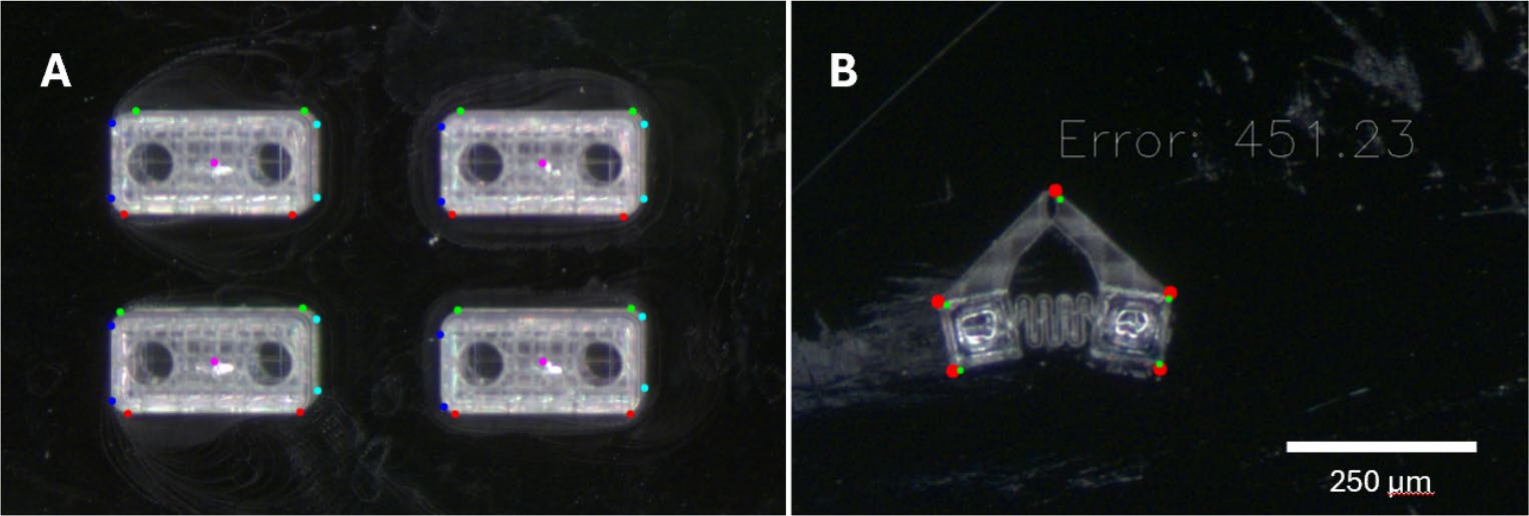
(**A**) Detection results for tumbling robots. (**B**) Detection results for gripping robots. Approximation in red, reference in green. Error units are pixels squared

### Detection of chassis

Two types of chassis detection schemes are used in this system, one for simpler geometry and one for more complex geometry. The simple method begins by taking an image, applying a binary color mask, and finding contours. Contours that fall outside an area threshold are discarded and the remaining contours are approximated as an octagon. An example of this is shown in Fig. 14(A). Two points that approximate one side of the chassis are shown in the same color. When chassis are oriented correctly, the line connecting green points should be horizontal, while that connecting blue should be vertical. The orientation of a chassis can be calculated based on the angle of these lines.

For more complex geometry, a more complex and robust algorithm is required. The algorithm used by this system is a simplified version of shape matching based on contour approximation. For this algorithm to work, an image of a computer model of the chassis in a desired orientation is used as a reference. The outline of the model is treated as a contour, which is dilated and eroded to simplify the geometry. This simplified contour is then approximated as a polygon, starting with three sides and increasing until the approximation error begins to change by only a small amount. After scaling and translation, the points of this polygon serve as reference points for a physical chassis in a desired location and orientation. To an image of one or more physical chassis, a color-based binary threshold is applied and contours are identified and filtered. Then the same steps as applied to the model can be applied to each contour in the image, yielding polygons with the same number of points as the reference polygon.

To obtain information about the position of the real chassis, the reference points must be matched as closely as possible to the approximation of the real chassis. This is done using the gradient descent optimization tool from the SciPy library. An error function is defined as the sum of squared distances between each reference point and its nearest real point. Then, a rotation and translation can be found that minimizes this error function. For a chassis with no radial symmetry there will be as many local minima as there are reference points. To prevent the algorithm from finding a non-global minimum, it iterates as many times as there are reference points after the initial optimization. Each iteration uses the translation found in the first optimization as an initial guess, and a range of initial rotation angles is used, evenly spaced from $0^{\circ }$ to $360^{\circ }$. This ensures that at least one repetition will converge towards the global minimum. The resulting optimal rotation and translation represent the rotational and translational deviation from the desired position, with an associated error in units of pixels squared. If optimization fails to converge below a certain error threshold, the detected object can be labeled as not being a chassis. An example of this algorithm is seen in Fig. 14(B), showing the estimated points, reference points, and error.

This detection scheme provides excellent accuracy and robustness, and consistent rejection of foreign bodies and noise. It also requires minimal computing resources and training data, requiring only a computer model and a single image of a real chassis. This makes it a robust detection algorithm for many different varieties of chassis.

### Detection of $\mu$-magnets

The system detects $\mu$-magnets in two possible states – grouped with other magnets or isolated. Magnets appear in the grouped state on the $\mu$-magnet stage, and in the isolated state on the assembly stage. Hence, different detection schemes are used for the different stages.

**Fig. 15:**
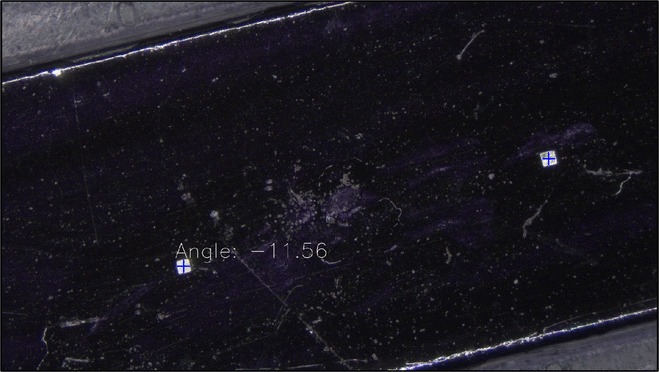
Detection results for isolated $\mu$-magnets. Blue crosses denote centroids. The angle shown is that of the leftmost magnet, in degrees

For the assembly stage, the goal is to identify every isolated $\mu$-magnet in view and find their orientations and locations. To do this, a color-based binary threshold is applied to a captured image of the stage with magnets on it. Contours in this image are identified, filtered by area, and dilated and eroded for smoothing. Using CV2 functions, the minimum area rectangle enclosing each contour is found. For a rectangle to be counted as a magnet, it must have sides within a certain size threshold, and it must have a percentage of filled area above a certain threshold. These filters effectively identify $\mu$-magnets’ locations and orientations and filter out noise from foreign objects and uneven surfaces. Results are shown in Fig. 15.

On the $\mu$-magnet stage, the goal is to identify the location of the $\mu$-magnet at the end of the group, as the tweezers need only grasp that magnet. The blue mark on the stage serves as an identifier to find the location of the anchor. Because the number of $\mu$-magnets is a known quantity, a mask can be applied to the image to only look at the area where magnets will be. A color-based threshold is applied to this mask and the resulting binary image is treated as a matrix. The first row with a sum above a preset threshold is treated as the Y position of the end of the magnet group; a similar procedure finds the X position. Surfaces of $\mu$-magnets are inconsistent in color, so sometimes they will be almost entirely masked out. If this occurs with the end magnet, the X result can be labeled as unreasonable and replaced with an X value based on the previous detection. The isolation process also straightens out the group of magnets, so after the initial isolation the Y position is highly consistent. This information is sufficient to consistently tweeze the end $\mu$-magnet.

### Tweezer detection

**Fig. 16:**
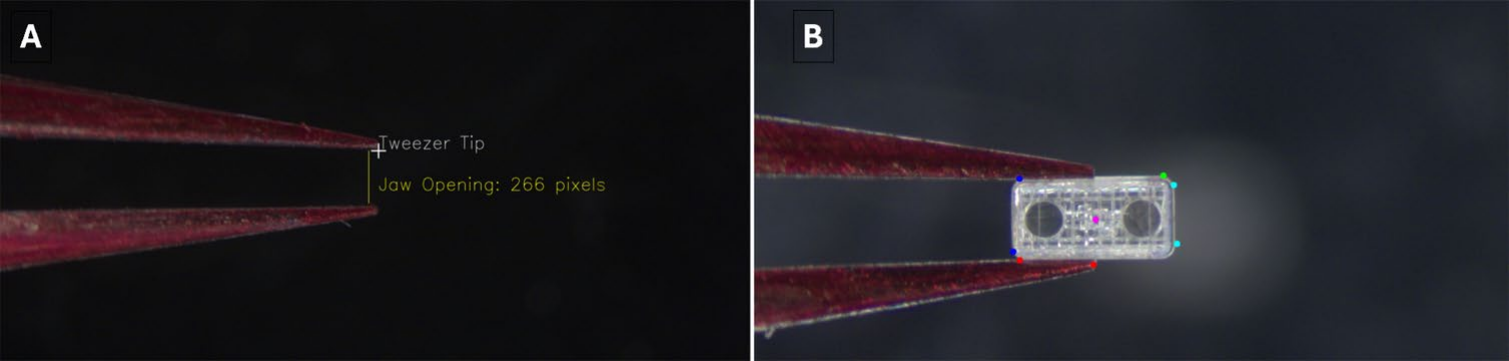
Tweezer detection. (**A**) Results of tweezer detection for calibration. (**B**) Estimation of a chassis being grasped

An image processing scheme for detecting the tweezers is also employed. Using color thresholding and contour detection and extracting extreme points from the contours, the position of the tweezers, as well as the jaw opening, can be found, as shown in Fig. 16(A). The upper arm is relatively stationary during actuation, so its tip is found and declared as the tweezer position. However, if the tweezers are grasping something, it interferes with this detection. This is circumvented by detecting the object being grasped rather than the tweezers themselves – an example of this is shown in Fig. 16(B). Despite an imperfect estimation of the chassis, the algorithm is robust enough to accurately find its center. This calibration is advantageous because the chassis center will be moved to a desired location when (5) and (6) are applied instead of the tweezers, which makes $\mu$-magnet embedding more accurate.

## Calibration and assembly results

### Calibration accuracy and timing

Table 1 presents data on the motion calibration. This data was taken by running the script 10 times without altering the configuration (altering the configuration can and should change the calibration results). This data demonstrates that the motion calibration is extremely precise. This is to be expected, since this calibration is entirely automated.

**Table 1:** Averages and standard deviations (SD) for motion calibration, taken over 10 trials. Units are pixels per micrometer (px/$\mu$m). $P_M$ is the directionless calibration factor. The other four variables are those from Eqs. 1 and 2. Time is the time taken to conduct the calibration

	$P_M$ (pixels/$\mu$m)	$X_X$ (px/$\mu$m)	$Y_X$ (px/$\mu$m)	$X_Y$ (px/$\mu$m)	$Y_Y$ (px/$\mu$m)	Time (s)
Average	0.339	−0.339	0.00936	−0.00769	−0.338	44.029
SD	0.000204	0.000204	0.000196	0.000425	0.000427	0.389

Data on the tweezer position calibration that is done during the process runtime is presented in Table 2. Once again, the data was taken by running the script 10 times without altering the configuration. This calibration only calibrates the X and Y coordinates, so no data for the Z axis is shown. Data is shown for both empty tweezers and for tweezers grasping a clean tumbler. The low standard deviations demonstrate the accuracy of the calibrations.

**Table 2:** Averages and standard deviations for position calibration. Results are shown both for empty tweezers and for tweezers grasping a tumbler, with 10 trials for each case

	Calibration with empty tweezers			Calibration holding tumbler		
	X ($\mu$m)	Y ($\mu$m)	Time (s)	X ($\mu$m)	Y ($\mu$m)	Time (s)
Average	10875.25	17775.62	5.39	9560.30	18085.50	6.75
SD	3.82	4.68	0.83	3.65	3.78	1.30

The Z axis calibrations are all done by hand, since they are simpler and require less time. The calibrations are read to the nearest micrometer. Tables 3 and 4 show data collected on the precision of the Z axis calibrations over 10 trials, and the times taken to perform them. These calibrations were also all performed without altering the configuration. Since the stage calibrations all follow the same procedure, they are presented as a single mean and standard deviation.

**Table 3:** Averages and standard deviations of Z axis calibrations, taken over 10 trials. The positions are the Z coordinates of the micromanipulator

	Stage ($\mu$m)	Embedding Needle ($\mu$m)	Injection tip ($\mu$m)	Wax brush ($\mu$m)
Average	9188	4364	9013	10198
SD	17	15	21	19

**Table 4:** Averages and standard deviations of time taken for Z axis calibrations, taken over 10 trials

	Stage (s)	Embedding Needle (s)	Injection Tip (s)	Wax brush (s)
Average	20.6	26.4	30.6	38.8
SD	3.9	5.1	3.2	7.7

The Sutter micromanipulator itself can also be recalibrated using its proprietary controller with its built-in calibration function. Based on the product information, the total drift of the coordinates is less than one micron per two hours, and the smallest resolution of microsteps available is 62.5 nanometers per step. Recalibration of the micromanipulator can be used to ensure the stability of the Z axis calibrations. This recalibration takes 2.69 seconds (SD = 0.11 seconds, n=10).

The frequency with which recalibration needs to be done is determined by how the system is used. While these calibrations can take time, provided there are no large disturbances to the system, there is no theoretical maximum number of assemblies that can be done without needing recalibration. The only part of the system that has a natural drift is the micromanipulator, and this can be calibrated independently of the rest of the system. Therefore, it is reasonable to assume 1 calibration at start up per batch of assemblies is sufficient. If the calibration time were to be included in the assembly time, it would be evenly distributed over all the assemblies and would decrease as the number of assemblies increases. Additionally, switching from embedding magnets in tumblers to embedding magnets in grippers only requires a single recalibration which will be done at startup. Therefore, calibration time is not reported as part of the robot assembly time.

### Control of wax seal deposition

**Fig. 17:**
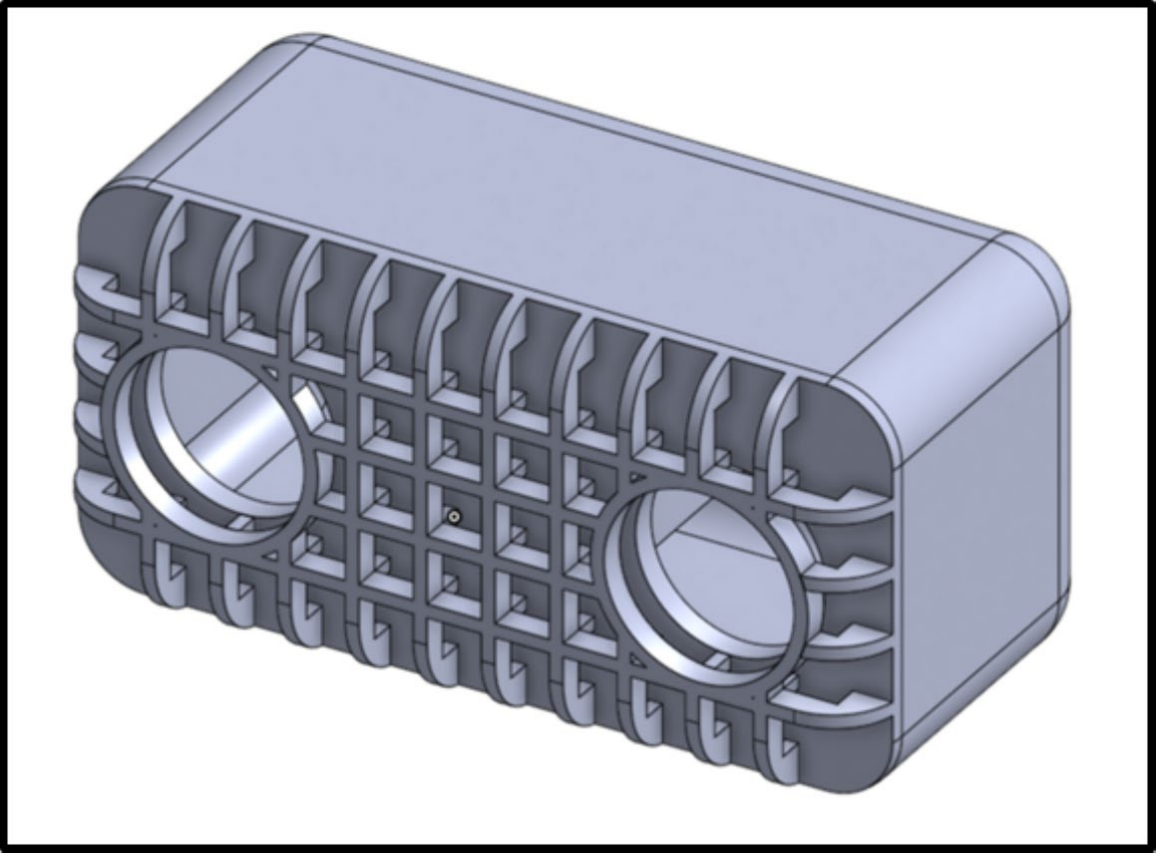
Altered robot design used for observing effect of temperature on wax seal deposition. The lack of side walls on the mesh allows an observer to see how the wax penetrates the mesh

For wax seals, the two most important aspects are that a seal is hermetic and adheres well to the robot. For a given robot design the properties of a seal can be influenced by changing temperature of the applied wax and the number of passes of the wax brush. To better observe the effects of variations in temperature, an altered robot design is used to allow better visibility and understanding of how the wax solidifies and adheres to the robot. This altered design is seen in the Fig. 17. The side walls of the mesh have been removed to afford this increased visibility.

**Fig. 18:**
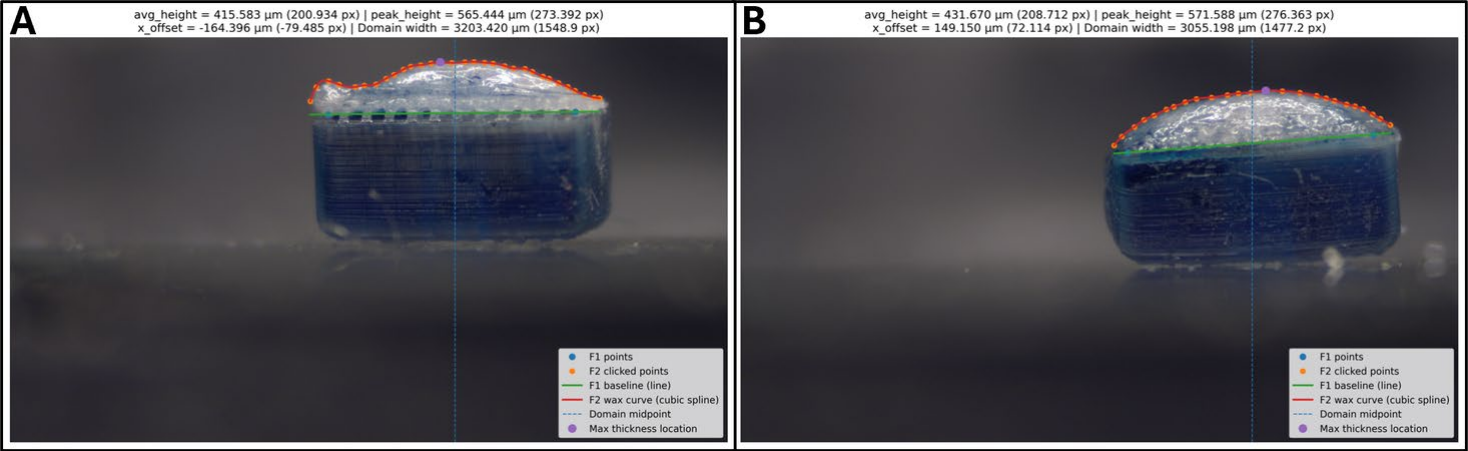
Images of the measurement program. (**A**) shows a seal deposited at $65^{\circ }\text {C}$, while (**B**) shows a seal deposited at $85^{\circ }\text {C}$. The seal in (**A**) visibly fails to fully penetrate the mesh and achieve a hermetic seal

Several trials were conducted at different temperatures using this altered design, which reveal distinct differences between the seal integrity and other properties. A few images showing how the seal properties are measured are shown in Fig. 18. We define a few variables based on these images for simplicity. Average thickness, in $\mu$m, is a measure of the average height of the deposited wax layer, and is calculated as the average of the vertical distance between the green line, which is considered as the base of the wax, and the red curve, which is considered the top of the seal. For seals where the wax does not fully penetrate the mesh, the baseline is defined by the observed bottom of the wax, not the base of the mesh. Max thickness in $\mu$m, is the greatest vertical distance between these two curves, denoted in the images as the lavender point. Peak deviation, in $\mu$m, is a measurement approximating the unevenness of the seal, and is defined as the difference between the maximum thickness and the average thickness. Peak offset, in $\mu$m, is a measure of how lopsided the seal is, and is defined as the horizontal distance between the point with maximum thickness and the horizontal center of the robot. Finally, there is a binary test to check the hermeticity of the seal, which is done by attempting to remove the liquid payload with capillary action, and visually confirming that there are no holes in the seal. For a set of tests at given temperature, the hermetic rate is defined as the percentage of seals that pass this test.

**Table 5:** Measured properties of wax seals for various temperatures. Results shown are mean±standard deviation

Wax temp (${}^{\circ }\text {C}$)	Number of samples	Average thickness ($\mu$m)	Max thickness ($\mu$m)	Peak deviation ($\mu$m)	Peak offset ($\mu$m)	Hermetic rate
65	6	434±32	615±78	181±46	108±95	16.67%
71	3	419±4	609±15	190±15	124±49	33.33%
77	4	460±37	633±48	173±19	224±83	100.00%
85	6	409±22	550±23	141±9	254±127	100.00%
100	3	341±16	471±18	130±5	408±36	100.00%

Table 5 shows measurements of different properties at different temperatures. Each test was performed with 10 passes on the wax brush. Several trends are noticeable in this data. The first is that the hermetic rate increases with temperature over the range of tested temperatures. This is the most important result from this data, and was the primary factor used in determining the operating temperature of the system. Since having hermetic seals is of paramount importance, data on other qualities of the seals need only be considered in temperature regions where hermeticity is consistently achieved. Based on the collected data, this occurs reliably between $77^{\circ }\text {C}$ and $100^{\circ }\text {C}$, although this range may be found to be larger from further testing. In this region, we note several trends that occur with increasing temperature: decreasing average thickness, decreasing max thickness, deceasing peak deviation, and increasing peak offset. Based on this data, $85^{\circ }\text {C}$ was chosen as the operating point for the system. This temperature was chosen based on a desire for seals with a low average thickness, peak deviation, and peak offset, but most importantly to be at a temperature with a wide margin of consistent hermetic rates. This results in seals that minimize changes to the shape of the robot while having strong adhesion and hermeticity.

**Table 6:** Measured properties of wax seals applied with various numbers of passes. Results shown are mean±standard deviation

Number of passes	Number of samples	Average thickness ($\mu$m)	Max thickness ($\mu$m)	Peak deviation ($\mu$m)	Peak offset ($\mu$m)	Hermetic rate
5	3	311±52	457±51	145±24	159±175	66.67%
10	16	302±25	408±33	106±12	129±62	100%
15	3	291±35	390±30	99±6	290±60	100%

Tests were also conducted to observe the effects of changing the number of passes of the wax brush. These tests were done with the original tumbler design, not the modified one. Table 6 presents the results of these tests. The hermetic rate at 5 passes is lower than at 10 or 15, likely due to the heating of the robot being less even, which results in less consistent and less evenly distributed seals. The standard deviations for 5 passes are all higher than those for greater number of passes, most likely for the same reason. Interestingly, robots sealed with 15 passes have a much greater peak offset than with 10 passes. For this reason and the greater hermetic rate, 10 passes was chosen to be the operating point of the system.

**Fig. 19:**
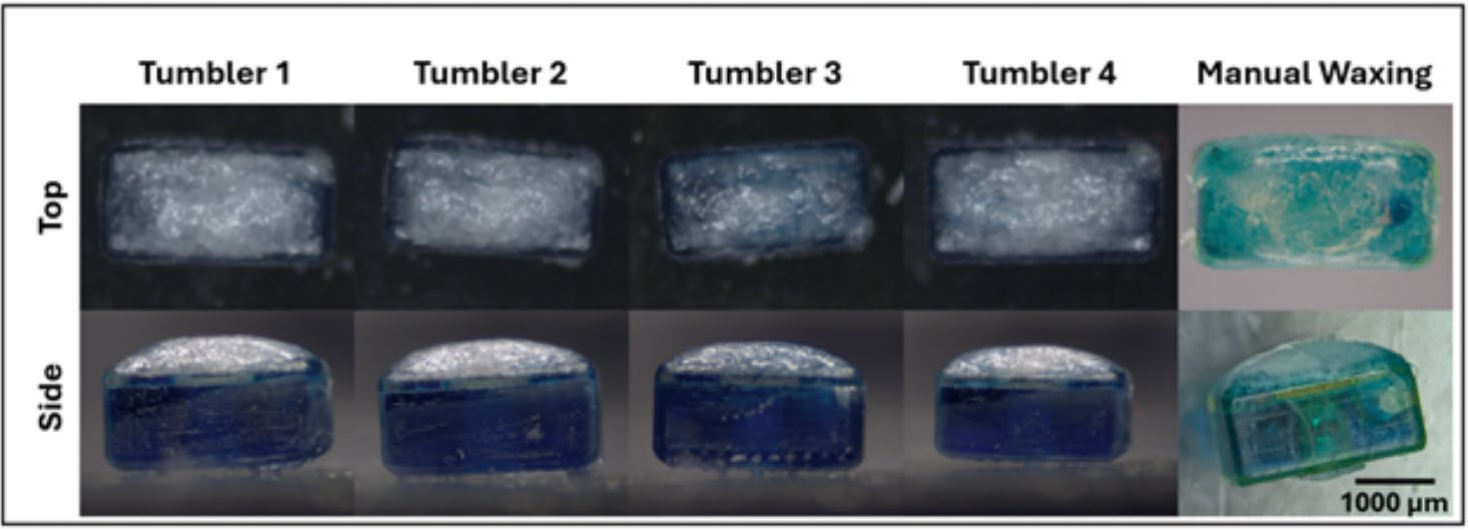
Four example pictures of filled and sealed tumblers, compared with a single example from the manual process

For system verification, 16 tumbler drugging and loading assemblies were done at the system operating point, with 10 brush passes at $85^{\circ }\text {C}$ on the un-altered robot design. The data from these tests is seen in Table 6. These samples passed the tests for hermetic sealing. The payload cavities were also slightly overfilled during the injection, proving that the sealing is effective for fully loaded microrobots. Some sample images of filled and sealed microrobots are provided in the following Fig. 19. All of these images clearly show a solid layer of wax over the entire top surface of the tumblers, covering the mesh and the drug ports.

One of the main benefits affored by this system is a significant reduction in the complexity of the drug loading and sealing process. The manual waxing process is far more complex, requiring that each robot be fully submerged in a melted wax bath twice and pressed with a hot razor blade to force the wax to make a proper seal [21]. Tweezers then need to be used to manually scrape off excess wax. The automated process makes this significantly simpler and faster. In addition to the time advantage, the increased coating consistency is a significant benefit. Although data is not available on the consistency of manually applied seals, it can be reasonable inferred that the automated process would be more consistent. Since wax is less dense than water, excessive wax coating can cause tumblers to float or become too buoyant, which makes them incapable of using a tumbling motion to navigate an aqueous environment.

### Process durations and failure rates

The time taken for the steps of different processes was also examined. The main source of variance in process times is calibration of the tweezer position and fixing of the jaw opening. Position calibration takes longer if tweezers or chassis are dirty, because there is more noise in the images. The amount of time needed to set the jaw opening is dependent on the initial condition of the jaw, which is uncertain when the system initializes. For this reason, magnet isolation, which is always the first step, has a greater variance. The variance from calibration could be reduced by conducting operations in a cleaner, more consistent environment, and the variance from setting the jaw opening could be reduced by the inclusion of an encoder on the tweezer actuator. Table 7 shows the averages and standard deviations for the times taken to complete different process steps, with the data being taken from 13 magnet embedding trials, and from 16 tumbler loading and sealing trials. Data from the embedding trials is divided into steps to better examine the sources of variance and make predictions about other assembly processes.

**Table 7:** Process durations for different assembly steps. Times shown are mean±standard deviation. Data is taken from a set of 13 trials for magnet isolation, chassis retrieval, shallow embedding, deep embedding, robot release, and full tumbler embedding. Data is taken from a set of 16 trials for full tumbler loading and sealing

Process	Process Time (s)
Magnet isolation	74.08±20.55
Chassis retrieval	29.69±6.79
Shallow embedding	29.77±11.47
Deep embedding	33.23±12.44
Robot release	16.00±3.56
Full tumbler embedding	192.77±48.28
Full tumbler loading and sealing	159.38±3.67

Using raw timing data from the tumbler magnet embedding experiments, a sample covariance matrix was computed for the individual process steps. Together with the mean step times, this matrix enables prediction of the mean and variance for assemblies that are linear combinations of the same steps.

The sample covariance between two steps $X_i$ and $X_j$ is computed as

8 $$\begin{aligned} \textrm{Cov}(X_i,X_j) = \frac{1}{n-1} \sum _{k=1}^{n} \left( X_{i,k}-\bar{X}_i\right) \left( X_{j,k}-\bar{X}_j\right) . \end{aligned}$$

For any assembly process expressed as a linear combination of steps

9 $$\begin{aligned} T = \textbf{w}^T \textbf{X}, \end{aligned}$$

the predicted mean and variance follow from standard linear propagation:

10 $$\begin{aligned} E[T]= & \textbf{w}^T \boldsymbol{\mu },\end{aligned}$$

11 $$\begin{aligned} \textrm{Var}(T)= & \textbf{w}^T \Sigma \textbf{w}. \end{aligned}$$

Predicted times and standard deviations are found in Table 8. Since the variance for tumbler loading and sealing is comparatively quite small, the full tumbler assembly process time variance is approximated as simply the sum of the variances of the embedding and loading/sealing processes instead of with Eq. 11. Variance for the gripper assembly, which is considered as two magnet isolations, one chassis retrieval, two shallow embeddings, and one robot release, is calculated with Eq. 11. Also included is the predicted assembly time for a microrobot containing four micromagnets and built with four magnet isolations, one chassis retrieval, four shallow embeddings, and one robot release. See Section 6 for further information on this microrobot.

**Table 8:** Predicted process times for gripper assembly and full tumbler assembly. Times shown are mean±standard deviation. Further information on the slithering robot can be found in Section 6

	Predicted process time (s)
Gripper assembly	273.38±68.86
Tumbler embedding, loading, and sealing	352.14±48.42
Slithering robot assembly	501.08±129.29

From real tests, the total time for gripper assembly is 315±4 seconds (n=2). These tests fall within a single standard deviation of the mean as predicted by the covariance matrix. For the 16 tumbler loading and sealing tests, no errors were observed. All samples were confirmed to be hermetically sealed. However, some errors were observed in the magnet embedding tests. Particularly, in 2 of the 13 tests, during the deep embedding step, the micromagnet being embedded stuck to the embedding needle and was pulled out from the chassis. This puts the failure rate for deep embedding at 15.4%. This failure requires the entire embedding process to be repeated from the beginning. It is likely that this failure rate would be significantly reduced if the embedding needle were made with a non-ferromagnetic material, which would help prevent the micromagnet from sticking to it. Based on this failure rate, it can be expected that on average, 2 in 13 embedding processes will need to be repeated, raising the average embedding process time from 192.77 seconds to 227.82 seconds. Other failure modes, such as failed micromagnet transportation from storage stage to assembly stage, failed chassis retrieval, and failed shallow embedding did not occur over any of the 13 trials. Precise timing for manually conducting these processes is unknown, but estimates from an operator who has conducted a small number of trials place the timing at approximately 420 seconds for a tumbler assembly, 600 seconds for a gripper assembly, and 450 seconds for loading and waxing a tumbler.

### Embedding results examples

**Fig. 20:**
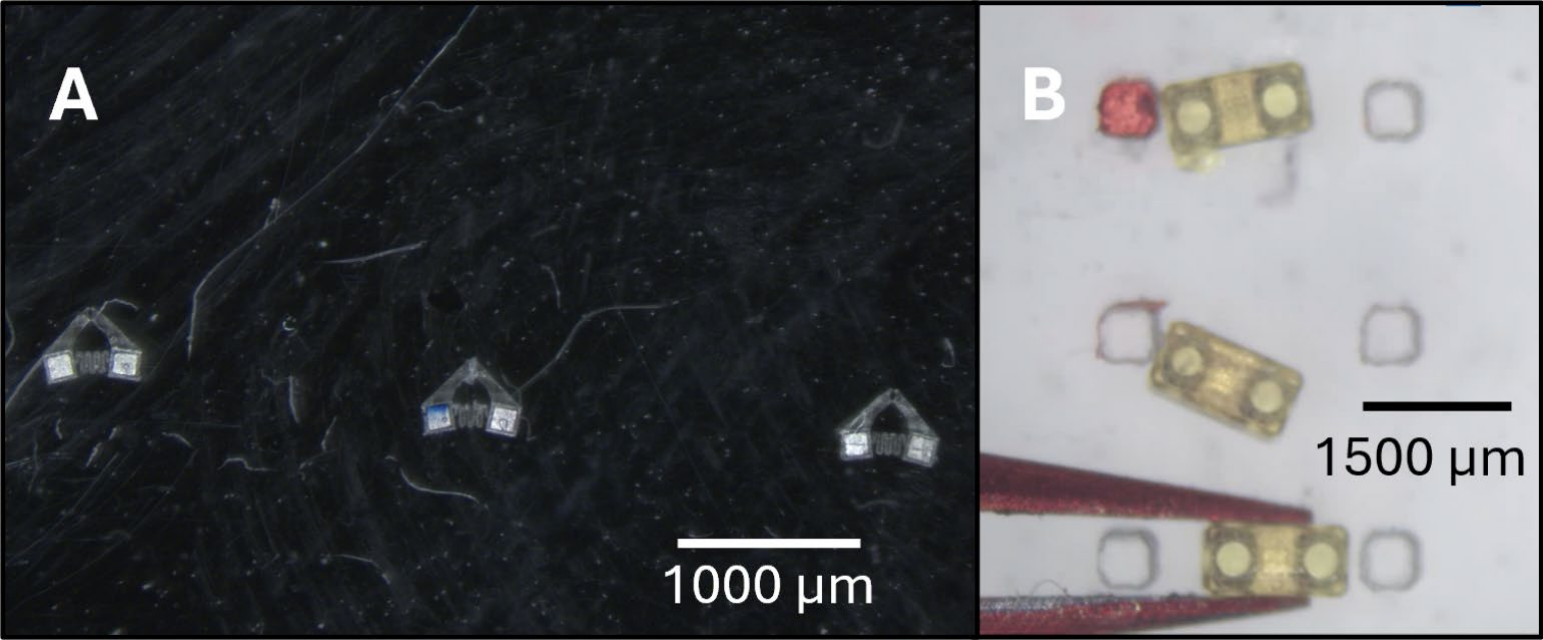
(**A**) Set of three grippers assembled by the system. (**B**). Three tumbling robots on the chassis stage after deep embedding, prior to washing or curing

Figure 20(A) shows a set of 3 gripping robots assembled by the system. They have been removed from the depot and washed. With the configuration presented in this paper, at most 10 grippers robots can be assembled in a single batch. Gripper assembly results are consistent and avoid damage to the chassis, which is a frequent issue in manual assembly, where failed assemblies often result in chassis and micromagnets being destroyed. The correct orientation of $\mu$-magnet poles is guaranteed by the automated assembly process.

Figure 20(B) shows two tumbling robots with magnets embedded on the robot depot, prior to washing or curing and without drug loading or waxing. The $\mu$-magnets embedded are less visible due to being further from the top surface of the chassis. The maximum number that can be assembled at once is again limited by the $\mu$-magnet storage capacity. At most 20 robots could be made in one batch with the current configuration.

## Current limitations and work extensions

**Fig. 21:**
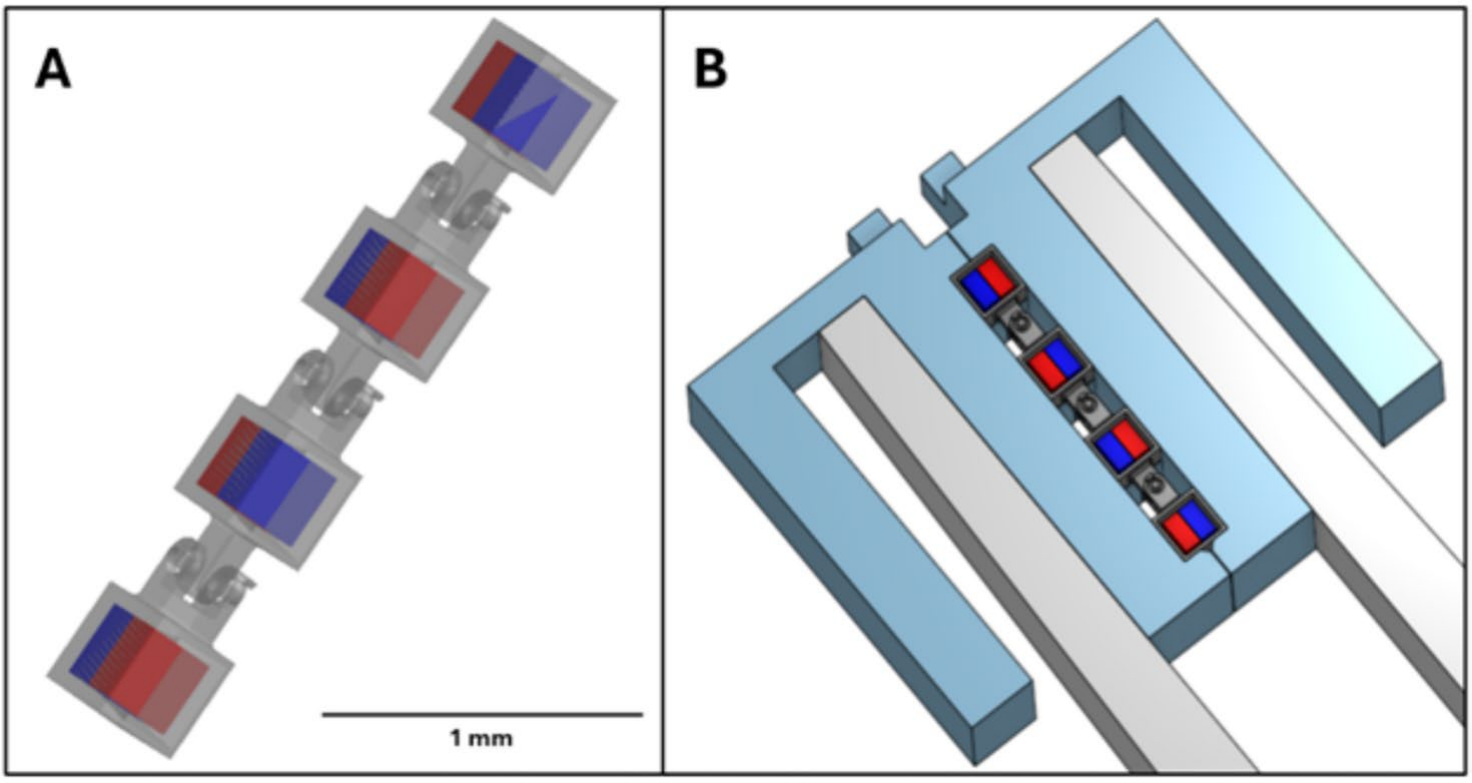
(**A**) Slithering robot design made of flexible PDMS, using four 250 $\mu$m-side embedded micromagnets. (**B**) Modified tweezer tips for grasping a slithering robot. These tips are also able to grasp micromagnets

A few geometrical factors can lead to some limitations of the system. The only part of the robot geometry that affects the assembly severely is the holes that the magnets are embedded into. A certain amount of interference fit is needed, as well as a certain amount of chamfering of the opening of the holes. These values have been empirically determined. The magnet as well must be the correct size to match the hole. The magnet dimensions are inspected ahead of using them in any assemblies. An additional restriction on these holes is that they must all have openings that lie on the same plane. Beyond this, the geometry of the robot makes little to no difference in embedding process. By modifying the tips of the tweezers, it is feasible to pick up nearly any 3D printed microrobot chassis that meets these criteria, and to insert the magnets as needed, even for very pliant chassis materials. Experiments have been done using 100 $\mu$m-side cube micromagnets that have shown promise. Figure 21 shows an example of an additional robot design that has been experimented with on the system, and new tweezer tips that can be used to grasp it. Building this robot would involve four magnet isolation steps, one chassis retrieval step, four shallow embedding steps, and one release step. Based on data from previous assemblies, it is predicted that this assembly would take 501.08±129.29 seconds. Other such robot designs could be similarly tooled to be made with the system.

**Fig. 22:**
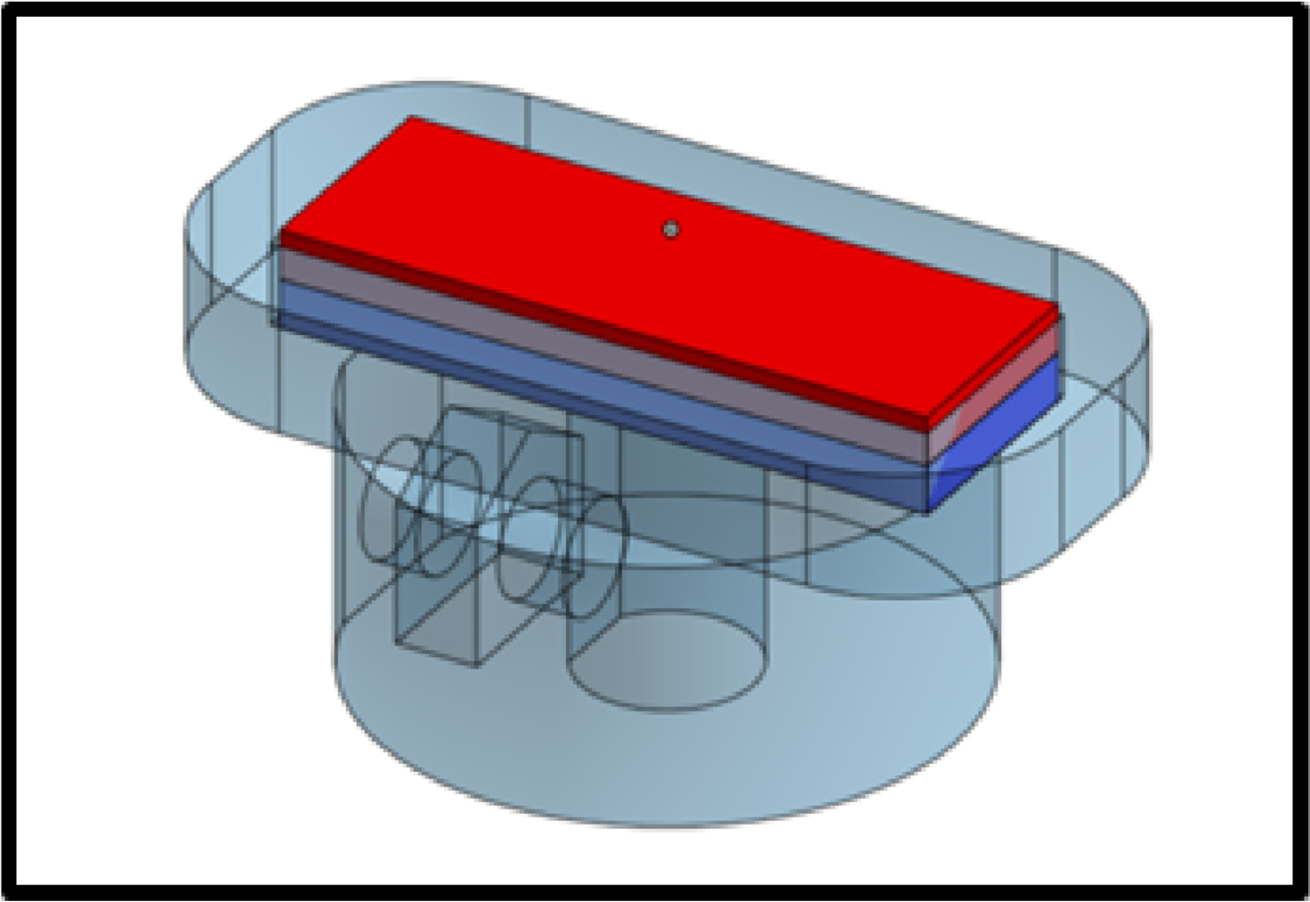
Vertically poled assembly stage concept for non-planar micromagnet assemblies

An extension to the existing system to accommodate robots with 3D magnetization profiles could be made by adding an additional assembly stage that is poled along the vertical axis. Figure 22 shows an example of how this could be done. Placing micromagnets on this stage would orient their poles vertically. They could then be assembled into robots in the same manner as is done on the existing assembly stage. Micromagnets to be assembled onto this stage could still be stored on the existing micromagnet storage stage. To accommodate vertical poling in the other direction, a third assembly stage can be used, or a mechanism can be installed to rotate the magnet in this stage about its long axis. It would also be possible to install a mechanism to rotate the tweezers about their own axis, albeit more difficult. However, an advantage would be provided by doing so, in that micromagnets could be assembled with their poling direction lying between entirely horizontal and entirely vertical.

The main limitations of the current system are batch size and throughput. The greatest batch size bottleneck that currently exists for the system is the number of micromagnets that can be loaded onto the system at once. The limit of micromagnet storage is currently 20 micromagnets. However, it is relatively easy to raise this quantity by either adding additional magnet anchors to the storage substage, or by adding additional storage stages. By increasing the size of the rotating main stage, it would be possible to add significantly more storage capacity for both micromagnets and robot chassis.

There are two modifications that could be made to increase the speed of the system. The first is to reduce the exposure time of the camera, which is set to 15 milliseconds. This exposure time makes it difficult to capture images of moving scenes. As a result of this, during magnet isolation, the micromagnet storage substage must move in discrete steps, pausing at set angles for the camera to acquire a still frame. If the exposure time was lower, the magnet isolation could be done in an approximately continuous manner. The second factor that is responsible for a lot of excess time is the rotation of the main stage. For one tumbler assembly, the system spends 46 seconds rotating the main stage, a number which is programmed into the system. The reason so much time is spent here is because of the quality of the motor used to rotate the base stage, and the feedback used to know precisely when the rotation is finished. Having a motor for this stage that could rotate the system faster and with less vibration could significantly reduce this time.

## Conclusion

This works presents the first system to fully automate the assembly of microrobots with embedded permanent magnets, and the complete loading and phase-change material sealing of drug delivery microrobots. This furthers progress towards scaling production quantity and makes feasible more microrobot designs that can make use of the benefits offered by embedded permanent magnets. It also offers a real solution to the large scale production of drug delivery microrobots with strong clinical potential, and advances the consistency with which they can be made. These results advance progress toward targeted drug delivery microrobots being used for clinical *in vivo* human use. Increasing batch size and throughput would be a valuable addition to a future version of this system. Future work may also consist of long duration locomotion tests at body temperature to further verify the robustness of the automated loading and coating procedure in real-world conditions.

## Supplementary Information

Below is the link to the electronic supplementary material.

Supplementary file 1 (mp4 88508 KB)

## Data Availability

No datasets were generated or analysed during the current study.
